# Transcriptome analysis of developing lens reveals abundance of novel transcripts and extensive splicing alterations

**DOI:** 10.1038/s41598-017-10615-4

**Published:** 2017-09-14

**Authors:** Rajneesh Srivastava, Gungor Budak, Soma Dash, Salil A. Lachke, Sarath Chandra Janga

**Affiliations:** 1 Department of Biohealth Informatics, School of Informatics and Computing, Indiana University Purdue University, 719 Indiana Ave Ste 319, Walker Plaza Building, Indianapolis, Indiana, 46202 USA; 2 Department of Biological Sciences, University of Delaware, Newark, DE 19716 USA; 3 Center for Bioinformatics and Computational Biology, University of Delaware, Newark, DE 19716 USA; 4 Center for Computational Biology and Bioinformatics, Indiana University School of Medicine, 5021 Health Information and Translational Sciences (HITS), 410 West 10th Street, Indianapolis, Indiana, 46202 USA; 5 Department of Medical and Molecular Genetics, Indiana University School of Medicine, Medical Research and Library Building, 975 West Walnut Street, Indianapolis, Indiana, 46202 USA

## Abstract

Lens development involves a complex and highly orchestrated regulatory program. Here, we investigate the transcriptomic alterations and splicing events during mouse lens formation using RNA-seq data from multiple developmental stages, and construct a molecular portrait of known and novel transcripts. We show that the extent of novelty of expressed transcripts decreases significantly in post-natal lens compared to embryonic stages. Characterization of novel transcripts into partially novel transcripts (PNTs) and completely novel transcripts (CNTs) (novelty score ≥ 70%) revealed that the PNTs are both highly conserved across vertebrates and highly expressed across multiple stages. Functional analysis of PNTs revealed their widespread role in lens developmental processes while hundreds of CNTs were found to be widely expressed and predicted to encode for proteins. We verified the expression of four CNTs across stages. Examination of splice isoforms revealed skipped exon and retained intron to be the most abundant alternative splicing events during lens development. We validated by RT-PCR and Sanger sequencing, the predicted splice isoforms of several genes *Banf1*, *Cdk4*, *Cryaa*, *Eif4g2*, *Pax6*, and *Rbm5*. Finally, we present a splicing browser Eye Splicer (http://www.iupui.edu/~sysbio/eye-splicer/), to facilitate exploration of developmentally altered splicing events and to improve understanding of post-transcriptional regulatory networks during mouse lens development.

## Introduction

The past decade has seen a surge in transcriptome-level studies for specific developmental stages of the eye and its tissue sub-types^1, 2^. The development of the eye involves a complex and highly orchestrated regulatory program with several specification and differentiation processes^3, 4^. The lens is a transparent tissue that focuses light on the retina^5^. It originates from the surface ectoderm early in embryogenesis and is composed of two cell types, namely the anteriorly located epithelial cells and the posteriorly located fiber cells^6, 7^. During development and throughout the life of the animal, epithelial cells differentiate into fiber cells that elongate and migrate towards the center of the lens, while degrading their organelles, including nucleus.

High-throughput sequencing techniques, collectively known as Next Generation Sequencing (NGS) approaches, have significantly advanced our understanding of the molecular portrait of various cell types and disease states^8–10^. One of the primary advantages of high-throughput RNA sequencing (RNA-Seq) is that it enables accurate assembly of the transcriptome, and its alterations across experimental conditions, so as to allow prioritization of the transcripts and splice forms that are potentially most relevant to the observed phenotype. However, employing RNA-Seq datasets for genome-scale elucidation of the splicing alterations across developmental^11^ and disease states^12^ or to study inter-individual differences in humans is still in its early stages^13^.

Greater than 94% of multi-exonic genes in the human genome are alternatively spliced^14^. Further, alternative splicing is an essential and highly controlled post-transcriptional regulatory mechanism which provides transcriptomic and proteomic diversity in eukaryotic organisms^15^. Due to the extensive prevalence of splicing events in higher eukaryotes, various transcriptomic datasets across developmental stages have been previously explored in multiple model organisms to study the structure and composition of protein-coding and non-coding genes^16–19^. These RNA-Seq based studies revealed more accurate and comprehensive set of known and novel genes for downstream functional and comparative analysis.

Previous studies report that ocular tissues such as the retina can exhibit highly diverse transcript profiles with hundreds of novel transcripts, likely contributed by the ensemble of multiple cell types abundant in retina^1, 20^. However, few RNA-Seq based studies have been conducted so far for investigating the lens transcriptome^21, 22^ especially over different developmental stages^23, 24^. Further, these studies have used only known or annotated genes in their analysis. Thus, to date the complete lens transcriptome and the various isoforms expressed in the developing lens has not been fully characterized. In this study, we investigated the transcriptomic alterations and splicing events from publicly available lens RNA-Seq data, and have constructed a comprehensive molecular portrait of known as well as novel transcript isoforms in the mouse lens across developmental stages.

## Results

**Figure 1:**
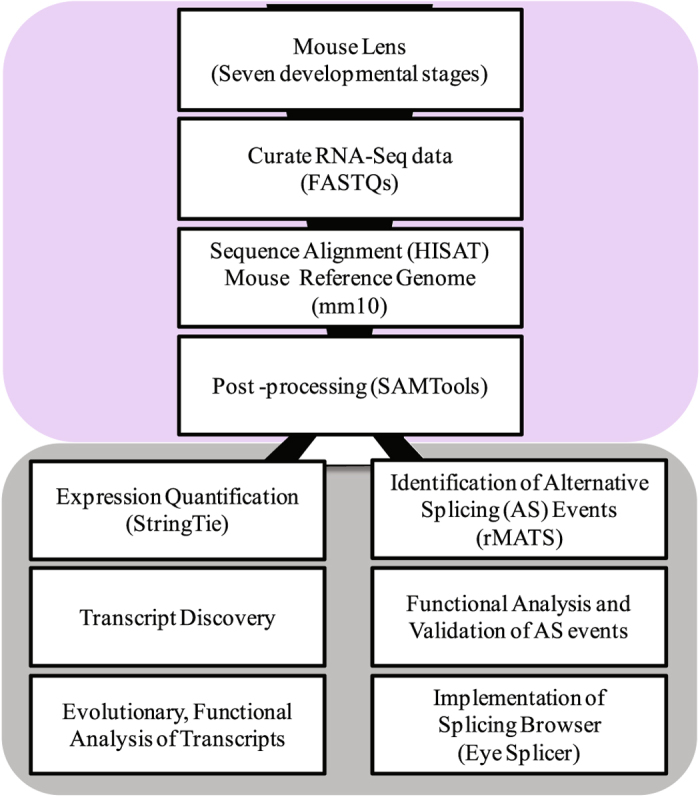
**(a**) Overview of the transcriptome analysis across developmental stages in mouse lens. Transcriptomes of mouse lens spanning seven developmental stages (three embryonic; E15, E15.5, E18 and four postnatal; P0, P3, P6, P9 stages with biological replicates) were collected from published sources for our study. Curated RNA sequence data was quality filtered using FASTX toolkit. High quality raw sequence reads were processed and aligned to mouse reference genome mm10 using HISAT and output collected as SAM files. Post processing (i.e. conversion of SAM to sorted BAM) of aligned reads was accomplished using SAMTools. Aligned and post processed RNA-Seq bam files associated with each developmental stage were utilized for two purposes. Firstly, for identifying and quantifying the expression levels of known and novel transcripts across seven developmental stages using StringTie, followed by an evolutionary and functional analysis to uncover high confident completely novel transcripts in developing lens. Secondly, the processed bam files were also employed for the identification of alternative splicing events using rMATS (replicate Multivariate Analysis of Transcript Splicing)^27^ followed by functional analysis of genes belonging to the enriched splice events. Finally, the results of the most prominent splicing events namely skipped exon and retained intron events are also made available through Eye splicer, a web based splicing browser showing developmentally altered splicing events in mouse lens.

Although mouse lens transcriptome profiling has been the focus of few studies in recent years^21–24^, our understanding of the complete repertoire of expressed transcripts and their splicing alterations during lens development is far from complete. In this study, we investigated the transcriptomic alterations and alternative splicing events in mouse lens across developmental stages. Overview of the analysis pipeline is illustrated in Fig. 1. In brief, we collected the available RNA-Seq data for mouse lens across varying developmental stages and processed the raw sequence reads using HISAT^25^ and StringTie^26^. The processed and quantified data were formatted into expression matrices and were utilized for investigation of complete transcriptomic architecture, extent of transcript novelty, and their evolutionary conservation (see Materials and Methods). Additionally, we investigated the alternative splicing events using rMATS^27^ followed by an extensive functional analysis of the genes associated with enriched splicing event types. The most prominent splicing event types namely skipped exon and retained intron events were made available through Eye splicer (http://www.iupui.edu/~sysbio/eye-splicer/), a web based splicing browser showing developmentally altered splicing events in mouse lens.

### Overview of the dataset and construction of the developmental transcriptomes in lens

We collected the RNA sequencing data from two studies^23, 28^ and preprocessed them using a NGS pipeline for data processing to facilitate their downstream analysis (See Materials and Methods, Fig. 1 and Table 1). The RNA-Seq data of different developmental stages were processed separately using the proposed pipeline. Raw RNA-Seq reads were aligned onto mouse reference genome mm10 using HISAT. The overall percentage of alignment for each sample is shown in Table 1. All datasets exhibited a good read quality (Phred score > 20) and a high fraction of read alignment to the reference genome (alignment score ≥ 93%) using HISAT.

**Table 1:** Metadata associated with the collected RNA-seq samples across lens developmental stages and results of their alignment with mouse mm10 reference genome from Ensembl.

S.NO	SRA IDs	D-Stage	PMID	Read Type	Read length	#Reads_Seq	#BaseCount	Overall %Alignment Rate
1	SRR2039769	E15	26225632	PE	100	13772390	2754478000	94
2	SRR2039770	E15	26225632	PE	100	13542500	2708500000	95
3	SRR953395	E15.5	24161570	SE	52	48552190	2524713880	94
4	SRR953394	E15.5	24161570	SE	52	47574424	2473870048	94
5	SRR953393	E15.5	24161570	SE	52	42525381	2211319812	94
6	SRR2039771	E18	26225632	PE	100	17810970	3562194000	93
7	SRR2039772	E18	26225632	PE	100	18019388	3603877600	93
8	SRR2039773	P0	26225632	PE	100	17766309	3553261800	93
9	SRR2039774	P0	26225632	PE	100	14533000	2906600000	93
10	SRR2039775	P3	26225632	PE	100	15495833	3099166600	93
11	SRR2039776	P3	26225632	PE	100	13072393	2614478600	93
12	SRR2039777	P6	26225632	PE	100	16965754	3393150800	93
13	SRR2039778	P6	26225632	PE	100	17658286	3531657200	93
14	SRR2039779	P9	26225632	PE	100	18874309	3774861800	93
15	SRR2039780	P9	26225632	PE	100	13563853	2712770600	93

**Figure 2:**
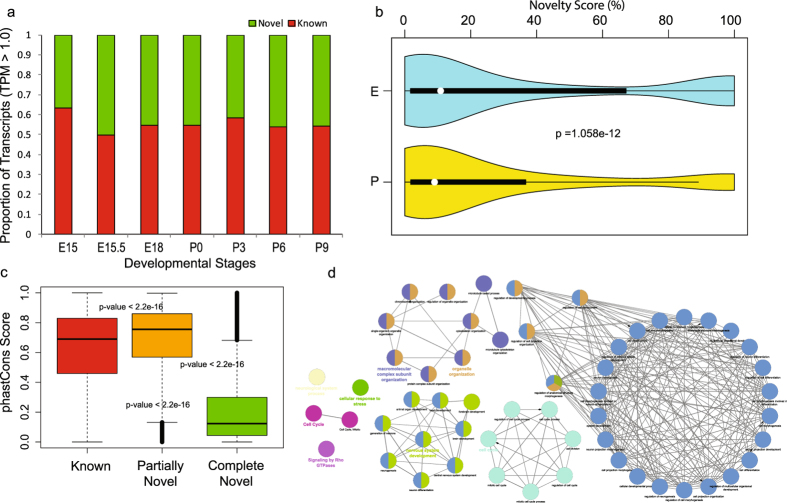
(**a**) Histogram showing the proportion of known and novel transcripts identified across various lens developmental stages in mouse. Only transcripts exhibiting an expression higher than 1 TPM (Transcripts Per Million reads sequenced) are considered in this plot. However, the proportions of known *versus* novel remained stable irrespective of the threshold on the expression level of a transcript (Figure S1). **(b)** Violin plot showing the distributions of novelty scores of identified transcripts, expressed in embryonic and postnatal stages. Violin plot represents the boxplot combined with kernel density showing the distribution pattern of a data vector. Novelty score of the transcripts expressed (with TPM > 5.0) at least in one stage were employed to generate two violin plots corresponding to the embryonic (E15, E15.5, E18) and postnatal (P0, P3, P6, P9) stages respectively. Differences in the distribution of novelty scores between embryonic and post-natal stages were compared using Kolmogorov–Smirnov test. Median novelty score for E and P were 10.89 and 9.043 respectively. **(c)** This panel shows the distribution of PhastCons scores, reflecting the extent of conservation for known, partially novel (novelty score < 70%) and completely novel (novelty score ≥ 70%) transcripts identified across developmental stages in lens. The phastCons score (PS) provides nucleotide level conservation of mouse genomic loci across 46 vertebrate genomes. We found each pair of these transcript classes to be significantly different in their extent of conservation (p < 2.2e-16, Wilcoxon rank sum test) with median conservation scores 0.67, 0.76, and 0.13 for known, partially novel and completely novel transcript groups respectively. (**d**) Gene ontology enrichment based functional grouping using annotations for genes corresponding to the high confidence partially novel transcripts (PS > 0.76). Functional grouping of the GO-terms based on GO hierarchy was represented as clustered GO-network using the Cytoscape^67^-ClueGO^31^ plugin. Significant clustering (p < 1e-10) of genes (color coded by functional annotation group they belong to) based on enriched GO-biological processes generated by ClueGO analysis, with size of the nodes indicating the level of significant association of genes per GO-term, were shown. Only selected biological processes and associated networks are shown in this figure panel, while Fig. S2 shows the complete set of functional groups identified from this analysis.

Since previous reports studying the eye transcriptomes indicated diverse transcriptomic architecture^4^, our goal was to investigate whether such diversity exists in different developmental stages of lens. For this purpose we first quantified the expression of transcripts and corresponding exons using StringTie. This allowed us to obtain expression levels for 90689 transcripts (68166 annotated and 22523 novel transcripts) in the mouse genome. The analysis indicated the existence of ~25% novel transcripts in the developmental mouse lens transcriptome. In order to further investigate the extent of the novel transcripts in each developmental stage, we analyzed the proportion of known and novel transcripts (with TPM > 1.0) across different developmental stages (Fig. 2a). We observed that in each of the developmental stages of mouse lens there are about ~35–50% of novel transcripts. Such variations in the distribution of known versus novel transcripts with respect to different developmental stages was found to be consistent despite filtering for different TPM thresholds (i.e. >0.5, >2.0, and >5.0). In particular, despite the expression threshold employed for defining the expression of a transcript, several thousands of novel transcripts were still identified (Figure S1a–c and Table S1). These observations support the presence of a diverse transcriptome with thousands of novel transcripts being expressed in various lens developmental stages as well as the predominance of complex transcriptional and post-transcriptional regulatory mechanisms in embryonic and post-natal stages during mouse lens formation.

### Embryonic stages exhibit the highest extent of novelty for the newly discovered transcripts with a significant decrease in post-natal stages

To further investigate whether the expression of these novel transcripts differs between stages, we calculated novelty score of a transcript to measure the differences in the extent of novelty across stages using KS (Kolmogorov–Smirnov) test. Novelty score of a transcript is defined as the percentage of non-overlapping novel transcript length to the reference annotated transcriptome (Fig. 2b). We observed that in embryonic stages, each pair of neighboring developmental stages were found to be significantly different in their distribution of novelty scores for the novel transcripts (p-value ≤ 0.005) and this pattern was observed until birth (P0). In general, the novelty score distributions of the novel transcripts for embryonic stages were observed to be significantly higher compared to those seen in post-natal stages (median novelty score: 10.89 *vs* 9.04, p = 1.06e-12, KS-test, Fig. 2b).

### Significant fraction of the partially novel transcripts in lens were found to be highly conserved across vertebrates and associated with neural system development, structural morphogenesis, protein localization, cell division and differentiation processes

In our study, we identified a total of 22523 novel transcripts (~25% of total transcripts) in mouse lens as documented in Table S2 along with their novelty score and expression levels. As discussed above, we observed differences in the distribution of novelty scores of transcripts between embryonic and postnatal developmental stages. Hence, we further classified the novel transcripts based on their novelty score (See Materials and Methods and Table S2). We categorized the novel transcripts into two groups; Partially Novel Transcripts (PNTs, novelty score < 70%, 13207 transcripts) and Completely Novel Transcripts (CNTs, novelty score ≥ 70%, 9316 transcripts).

To investigate and compare the extent of conservation of known and novel transcripts, we used phastCons scores from UCSC Genome Browser, which provide a nucleotide level conservation score across 46 vertebrate genomes, facilitating a measure to quantify conservation for mouse genomic loci (see Materials and Methods). We calculated the phastCons score distributions for each group of transcripts; known transcripts, PNTs and CNTs (Materials and Methods section, Fig. 2c). We observed a significant difference in phastCons score distributions among these groups (median for known transcripts = 0.67, median for PNTs = 0.76, and median for CNTs = 0.13; Wilcoxon rank sum test, p-value < 2.2e-16). The score distribution indicates that PNTs exhibit higher conservation patterns than already known transcripts while their patterns were less comparable to CNTs. These observations suggest that since lens tissue and corresponding cell line transcriptomes have been poorly or rarely studied by genome annotation consortiums like ENCODE^29^ or FANTOM^30^, it is possible that hundreds of transcripts specific to lens may have been rarely documented in genomic/transcriptomic resources. However, integrative analyses and databases based on next generation RNA-sequencing datasets specific to such overlooked tissues, would be able to capture such missing transcript isoforms or poorly annotated genes, suggesting the need for such focused studies. In contrast, most of the CNTs were found to be poorly conserved based on phastCons score profiles. Interestingly, we found a few of the CNTs as outliers in the box plot exhibiting extremely high conservation (Fig. 2c, CNTs, above third quartile), which met the median phastCons threshold of both known and CNTs, and hence are likely to be active but functionally uncharacterized for biological processes.

To understand whether particular functions and processes are over-represented as gene ontology (GO) categories for these novel transcripts, we performed functional enrichment analysis of the PNTs by using the annotations of the corresponding mouse genes with which they overlap partially. To generate a high confident set of evolutionary conserved novel transcripts with annotated information, we filtered the PNTs with phastCons score > 0.8 and obtained a set of 3982 genes satisfying these criteria. We performed functional enrichment analysis of these genes with corrected p-value (Bonferroni correction) threshold < 10^−10^ using ClueGO^31^. ClueGO is a Cytoscape plugin which enables the functional grouping of GO terms or gene sets to represent the enriched functional themes as networks. Figure S2 shows the resulting network for GO biological processes. We found significant clustering of genes into 26 thematic groups based on enriched GO terms using ClueGO (Table S3). Specific biological processes and associated modules are highlighted in Fig. 2d. We observed that ‘alternative mRNA splicing via spliceosome’, ‘mRNA metabolism process’, ‘ubiquitin mediated proteolysis’, ‘nervous system development’, ‘neurological system process’, ‘organelle organization’, ‘cell cycle’, ‘protein localization’ etc were over-represented in PNTs (Fig. 2d and Figure S2). For instance, we found group 19 (i.e. nervous system development) to be significantly enriched (adjusted p-value = 9.93e-32) with 841 genes i.e. ~30% of the genes (Table S3) annotated with neurogenesis, neuron differentiation and nervous system developmental processes. These observations clearly reveal the role of several poorly characterized transcripts associated with nervous system development, RNA metabolism, cell cycle, organelle and chromatin organization, regulation of anatomical structure morphogenesis and cell differentiation, during lens development.

### Majority of the complete novel transcripts are widely expressed across developmental stages albeit exhibiting significantly lower expression, conservation and length compared to partially novel transcripts

We further investigated and compared the transcriptomes of the three groups of transcripts across each developmental stage. We averaged the expression level of a transcript across biological replicates in each developmental stage in order to compare the distribution of expression levels for known transcripts, PNTs and CNTs. We included the subset of transcripts in each class which were found to be expressed in all seven stages which resulted in 23121 known transcripts, 4531 PNTs and 4027 CNTs. The expression values were log-transformed and represented as box plot for each class across individual stages separately (Figure S3). We observed that, all three transcript classes exhibited significantly different expression profiles for each developmental stage (Wilcoxon rank sum test, p-value < 0.001, See Table S4), with known and PNTs exhibiting significantly higher expression compared to CNTs. In particular, our analysis also revealed that PNTs are highly expressed than known transcripts (Wilcoxon rank sum test, p-value < 0.001, See Table S4). These observations are similar to the conservation pattern of PNTs being higher than other transcript groups. These results indicate that PNTs are significantly more expressed than CNTs across all developmental stages and are often more expressed than even annotated transcripts suggesting that these PNTs are likely functional in lens development.

**Figure 3:**
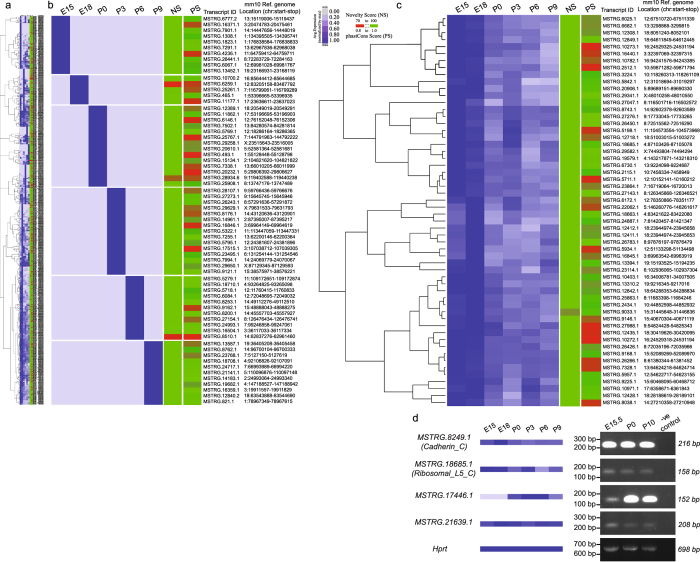
Completely novel transcripts (CNTs) with high conservation score (phastCons Score > 0.8), and expressed in atleast one developmental stage are shown across the panels. Expression profiles are normalized by the maximum expression level of a given transcript across stages and hierarchically clustered using Cluster 3.0 and visualized as a heatmap using Java Treeview. Samples from E15.5 that came from a different study than the rest of the samples were excluded from this expression analysis in order to avoid the batch effect. Heat maps showing the expression profiles of (**a**) 647 completely novel (novelty score ≥ 70%) transcripts hierarchically clustered with representative transcript groups expressed (**b**) in only one specific developmental stage and (**c**) in all the developmental stages. Novelty score (NS) and phastCons score (PS) indices for transcripts are also shown in as an additional scale bar in each heat map. (**d**) RT-PCR analysis validates expression of two CNTs with a predicted ORF (*MSTRG.8249.1* and *MSTRG.18685.1*) and two CNTs with no known ORF (*MSTRG.17446.1* and *MSTRG.21639.1*) in E15.5, P0 and P10 lenses. Note that *MSTRG.17446.1* is undetected in this analysis at stage E15.5. *Hprt* represents a loading control. Negative control is included for all CNTs tested where the RT-PCR reaction was performed using the same primers as for the CNTs but without any cDNA. Full-length gels are included in Supplementary Information file.

Although we observed that transcripts belonging to the CNT class were generally poorly conserved compared to the other two groups (Fig. 2c), nevertheless a small fraction (~8.6%) of CNTs exhibited high conservation with phastCons scores greater than 0.76. We considered these CNTs as highly conserved completely novel transcripts because the median conservation score of known and partially novel transcripts was found to be 0.67 and 0.76 respectively. In order to further interrogate the activity of these ~8.6% completely novel transcripts, we analyzed their expression profile across developmental stages. We further filtered them to obtain a set of CNTs with a phastCons score > 0.8 and expressed in at least one developmental stage, after excluding RNA-seq samples from E15.5 which originate from a different study in order to avoid any potential batch effect. We found a total of 647 CNTs (see Table S5) that exhibited varying levels of expression across developmental stages (Figure S4). Figure 3a shows a clustering snapshot of the distribution of these expression profiles across stages with expression levels of a transcript normalized by its maximum level across developmental stages (Materials and Methods, see Figure S4 for an extended heatmap). We analyzed the expression profiles of CNTs based on hierarchical clustering to identify representative panels of transcripts expressed in only one specific developmental stage (Fig. 3b) and in all developmental stages analyzed (Fig. 3c). These heatmap panels show the genomic co-ordinates as well as the novelty and phastCons scores associated with each CNT. We observed that ~10% of the CNTs (phastCons score > 0.8) were expressed in specific developmental stages as shown in Fig. 3b. In contrast, ~47% of the transcripts were found to be expressed across all developmental stages, with a selected set of hierarchically clustered CNTs following this trend shown in Fig. 3c. This suggests that a small fraction of CNTs with uncharacterized function could be potentially regulating stage specific developmental processes while majority of the CNTs could have broader functional roles across stages albeit uncharacterized.

We also investigated the transcript structure of different transcript classes by comparing the number of exons and length distributions. We observed significant difference in the distribution of exonic composition for PNTs and known transcripts (p < 2.2e-16, Kolmogorov–Smirnov test), with majority of the PNTs being multiexonic (>3 exons). In particular, about 20% of the PNTs were found to have more than 20 exons and were enriched in genes associated with several processes including ‘microtubule cytoskeleton organization’, ‘cell cycle’, ‘nervous system development’, ‘cell projection morphogenesis’, ‘embryo development’, ‘focal adhesion’ and ‘chromatin remodeling’. In contrast, we observed that ~90% of CNTs were single or bi-exonic with a small fraction of them exhibiting multiexonic structure as shown in Figure S5a. We also investigated the length for the three groups of transcripts and found significantly (p-value < 2.2e-16) varying distribution of lengths as shown in Figure S5b. We observed that the known transcripts exhibited an expected distribution of transcript length as previously described^32^ with an abundance of transcripts having length between ~10^2^ bp and ~10^4^ bp. However, among the novel transcript groups; PNTs exhibited a distribution more similar to that of known transcripts when compared to CNTs. In particular, we observed that most PNTs had length ranging from 10^2^–107 bp with abundance of transcripts having length in the range of 10^5^–10^6^ bp. In contrast, we found majority of the CNTs ranging in length from 10^2^ to 10^5^ bp dominated by relatively shorter length (100–1000 bp) transcripts. Indeed, studies from GENCODE consortium^33^ observed that human long noncoding RNAs (lncRNAs) are typically encoded as single or biexonic transcripts with significantly lower exome lengths compared to annotated protein coding transcripts, suggesting that several of the CNTs detected in our study are likely to be noncoding RNAs.

We hypothesized that the varying exonic composition and transcript length distribution of CNTs could help further filter them in order to build a high confidence compendium of active CNTs for downstream analysis. We thus included transcript length as an additional parameter along with high conservation score to delineate probably functionally active 100% novel transcripts. We applied two sets of filters; a) 300 bp ≤ transcript length ≤ 10000 bp; phastCons score > 0.95; average expression > 5.0 TPM and expressed in at least four developmental stages and b) 300 bp ≤ transcript length ≤ 10000 bp; phastCons score > 0.95; no expression threshold; but expressed in at most two developmental stages to generate two sets of CNTs. Table 2 show a list of 25 and 19 CNTs satisfying these two sets of filters respectively. To investigate if any of the highly conserved 25 CNTs can encode for potential functional protein coding sequences, we performed ORF prediction using an ad hoc Python script to detect both canonical and non-canonical start codons in six open reading frames. This analysis allowed us to confirm the presence of ORFs in 13 CNTs. Among these 13 ORFs, 8 of them were found to contain Pfam domains according to HMMER^34^ (Table S6). We performed ORF prediction on 654 CNTs that are 100% novel and exhibited a phastCons score > 0.8. We observed that 202 of them encode for ORFs with 121 of them exhibiting at least one hit using HMMSCAN^34^ against Pfam, suggesting that at least 18% of the CNTs are likely to encode for functional domains (Table S7). Further, we validated four of the CNTs shown in Table 2 by RT-PCR in three different developmental stages of lens, among which two transcripts were predicted to encode for ORFs (Fig. 3d, Figure S6). We found that all the four completely novel transcripts were expressed in P0 and P10 stages. As predicted from our transcriptomic analysis, the MSTRG.17446.1 transcript was not detected at E15.5. These results further validate the stage-specific expression of CNTs shown in Fig. 3 and Table 2

**Table 2:** High confidence list of 100% novel transcripts along with their genomic co-ordinates, length, strand and phastCons score.

Gene ID	Transcript ID	Coordinates (mm10)	Length (bp)	Strand	phastCons Score	Novelty Score
MSTRG.10752	MSTRG.10752.1	16:91113662-91114155	493	.	0.997	100
MSTRG.11071	MSTRG.11071.1	17:16557283-16557630	347	.	0.997	100
MSTRG.11709	MSTRG.11709.2	17:39636629-39637143	514	−	0.997	100
MSTRG.13417	MSTRG.13417.2	19:17926989-17927998	1009	+	0.993	100
MSTRG.9039	MSTRG.9039.1	15:31655084-31655385	301	.	0.990	100
MSTRG.17446	MSTRG.17446.1	3:104752371-104752702	331	−	0.989	100
MSTRG.30048	MSTRG.30048.1	X:146176602-146177068	466	+	0.989	100
MSTRG.12849	MSTRG.12849.1	18:64611849-64612445	596	−	0.989	100
MSTRG.1816	MSTRG.1816.1	1:178299808-178300378	570	+	0.987	100
MSTRG.18663	MSTRG.18663.1	4:83421622-83422080	458	−	0.987	100
MSTRG.6455	MSTRG.6455.1	12:102866597-102867299	702	.	0.984	100
MSTRG.12840	MSTRG.12840.1	18:63543865-63545205	1340	+	0.981	100
MSTRG.21639	MSTRG.21639.1	5:127954976-127955377	401	.	0.978	100
MSTRG.8143	MSTRG.8143.1	14:33428605-33429024	419	.	0.973	100
MSTRG.13495	MSTRG.13495.1	19:25346816-25347223	407	.	0.973	100
MSTRG.15865	MSTRG.15865.1	2:157528311-157528617	306	.	0.970	100
MSTRG.20543	MSTRG.20543.1	5:46415104-46415426	322	+	0.970	100
MSTRG.6330	MSTRG.6330.1	12:86845755-86846203	448	.	0.970	100
MSTRG.8249	MSTRG.8249.1	14:48695580-48695978	398	.	0.969	100
MSTRG.7535	MSTRG.7535.1	13:91260953-91262729	1776	+	0.969	100
MSTRG.18684	MSTRG.18684.1	4:87103424-87105078	1654	+	0.967	100
MSTRG.18685	MSTRG.18685.1	4:87103426-87105078	1652	−	0.967	100
MSTRG.6715	MSTRG.6715.1	13:8289064-8289370	306	.	0.966	100
MSTRG.7905	MSTRG.7905.1	14:15949235-15949774	539	.	0.965	100
MSTRG.7913	MSTRG.7913.1	14:16451876-16452185	309	.	0.953	100
**Gene ID**	**Transcript ID**	**Coordinates (mm10)**	**Length (bp)**	**Strand**	**phastCons Score**	**Novelty Score**
MSTRG.16504	MSTRG.16504.1	3:36117033-36117334	301	.	1.000	100
MSTRG.18476	MSTRG.18476.1	4:53663579-53663885	306	.	0.993	100
MSTRG.12840	MSTRG.12840.2	18:63543888-63544690	802	+	0.989	100
MSTRG.444	MSTRG.444.1	1:52585218-52585601	383	.	0.988	100
MSTRG.7901	MSTRG.7901.1	14:14447656-14448019	363	.	0.982	100
MSTRG.25445	MSTRG.25445.1	7:127398616-127399037	421	.	0.980	100
MSTRG.3390	MSTRG.3390.1	10:129665538-129665871	333	.	0.975	100
MSTRG.6084	MSTRG.6084.1	12:72048695-72049032	337	.	0.975	100
MSTRG.10567	MSTRG.10567.1	16:53985023-53985331	308	.	0.973	100
MSTRG.10245	MSTRG.10245.1	16:23050282-23050873	591	.	0.973	100
MSTRG.6777	MSTRG.6777.2	13:15119006-15119437	431	+	0.970	100
MSTRG.26441	MSTRG.26441.1	8:72283729-72284163	434	.	0.969	100
MSTRG.10714	MSTRG.10714.1	16:88682165-88682703	538	.	0.966	100
MSTRG.25152	MSTRG.25152.1	7:107951379-107951776	397	.	0.963	100
MSTRG.14539	MSTRG.14539.1	2:34648981-34649511	530	−	0.956	100
MSTRG.6778	MSTRG.6778.2	13:15118406-15118934	528	+	0.956	100
MSTRG.13452	MSTRG.13452.1	19:23166931-23168119	1188	.	0.956	100
MSTRG.12890	MSTRG.12890.1	18:67920731-67921163	432	.	0.955	100
MSTRG.13017	MSTRG.13017.1	18:90311445-90311766	321	.	0.952	100

High confidence list of transcripts with transcript length (> 300 & < 10000 bp), phastCons score > 0.95; and average expression > 5.0 TPM in atleast four developmental stages. High confidence list of transcripts unique to developmental stages with transcript length (>300 & < 10000), phastCons score > 0.95; expressed (no exp. threshold, as most of transcripts in this category are poorly expressed) in at most two developmental stages.

.

### Splicing analysis reveals abundance of skipped exons and retained intron events across developmental stages

Alternative splicing is an important molecular mechanism which contributes to the transcriptomic diversity in higher eukaryotes^35^. Increasing evidence supports the role of splicing and post-transcriptional regulatory alterations in development^11^ and disease^12, 36–38^, in addition to their prominent role in generating multiple transcripts and protein isoforms in normal cells.

Since we observed significant differences in the distribution of novelty scores for novel transcripts between the embryonic and post-natal stages in mouse lens, we argued that alternative splicing could contribute to these differences. In addition to contributing to transcript isoforms, splicing events can also contribute to differential regulation of the gene products across developmental stages by controlling the abundance of the required isoform. Hence, we employed rMATS^27^, a framework for detecting splicing alterations from next generation RNA-sequencing datasets, to investigate such key events for molecular diversity across developmental stages (see Materials and Methods). Table 3 shows the number of high confident Alternative Splicing (AS) events detected using rMATS pipeline (FDR < 0.01) across every pair of developmental stages with replicates. Table includes the number of detected AS events reported to be significant by rMATS, for the five types of events namely skipped exon (SE), alternative 5′ splice site (A5SS), alternative 3′ splice site (A3SS), mutually exclusive exons (MXE) and retained intron (RI). These results clearly indicate an abundance of SE and RI events compared to the other types during lens development. Tables S8 and S9

**Table 3:** Identification of alternative splicing events using rMATS (replicate Multivariate Analysis of Transcript Splicing)^27^.

AS Events Summary	E15 vs E18	E15 vs P0	E15 vs P3	E15 vs P6	E15 vs P9	E18 vs P0	E18 vs P3	E18 vs P6	E18 vs P9	P0 vs P3	P0 vs P6	P0 vs P9	P3 vs P6	P3 vs P9	P6 vs P9
SE	7	52	40	55	55	120	75	123	87	10	9	14	3	5	0
MXE	3	6	18	8	12	6	16	4	10	14	2	3	10	14	0
RI	62	46	25	26	37	73	29	31	34	6	2	9	0	1	2
A5SS	1	9	0	4	0	7	0	2	0	1	1	2	0	0	0
A3SS	3	4	3	3	3	12	5	7	5	1	2	0	1	0	1
**Exon Skipping Events**															
**Exon ID**		**Coordinates (mm10)**		**strand**		**Transcript ID**		**Gene Name**		**E15**	**E18**	**P0**	**P3**	**P6**	**P9**
ENSMUSE00000334942		19:57051130-57051234		−		ENSMUST00000111559		Ablim1		NA	0.13	0.61	0.729	0.66	0.612
ENSMUSE00000668725		3:148849766-148849804		−		ENSMUST00000197567		Adgrl2		0.202	0.24	0.61	NA	0.57	0.611
ENSMUSE00001324776		1:82891460-82891507		+		ENSMUST00000190052		Agfg1		0.232	0.18	0.41	0.532	0.48	0.489
ENSMUSE00001039657		18:6057517-6057591		−		ENSMUST00000182066		Arhgap12		0.289	0.32	0.82	NA	NA	NA
ENSMUSE00000700987		2:10056770-10056806		−		ENSMUST00000114897		Atp5c1		0.822	0.76	0.55	0.55	0.62	0.574
ENSMUSE00000230008		18:32426224-32426352		+		ENSMUST00000091967		Bin1		NA	0.68	0.18	NA	NA	NA
ENSMUSE00000217920		9:70004306-70004341		+		ENSMUST00000034754		Bnip2		NA	0.5	0.9	0.806	NA	0.845
ENSMUSE00000736151		10:127064202-127064453		+		ENSMUST00000133115		Cdk4		0.959	0.96	0.8	NA	NA	NA
ENSMUSE00000691476		5:112251747-112251797		−		ENSMUST00000112385		Cryba4		0.974	0.98	NA	0.994	0.99	0.99
ENSMUSE00000311733		14:47726471-47726554		+		ENSMUST00000022391		Ktn1		NA	0.24	0.5	0.476	NA	0.517
ENSMUSE00000440236		6:93680789-93680877		−		ENSMUST00000204347		Magi1		0.118	0.09	NA	NA	0.48	0.655
ENSMUSE00000667965		7:143518850-143518885		−		ENSMUST00000072727		Nap1l4		0.477	0.42	0.29	0.228	0.25	0.245
ENSMUSE00001311933		2:105695306-105695456		+		ENSMUST00000111082		Pax6		0.995	1	0.94	0.993	1	NA
ENSMUSE00000317905		15:93452117-93452173		+		ENSMUST00000068457		Pphln1		0.151	0.07	0.57	0.356	0.5	0.639
ENSMUSE00000635082		9:86790056-86790139		−		ENSMUST00000074468		Snap91		NA	0.85	0.27	0.248	0.2	NA
ENSMUSE00001196118		11:80393084-80393176		+		ENSMUST00000123726		Zfp207		0.368	NA	0.67	NA	0.61	0.593
**Intron Retention Events**															
**Exon ID**		**Coordinates (mm10)**		**strand**		**Transcript ID**		**Gene Name**		**E15**	**E18**	**P0**	**P3**	**P6**	**P9**
ENSMUSE00000784872		3:103174340-103177419		+		ENSMUST00000136937		Bcas2		0.039	0.0685	0.026	0.035	0.0225	0.031
ENSMUSE00000787504		11:101295534-101296316		−		ENSMUST00000139997		Becn1		NA	0.1185	0.0635	0.062	0.051	0.044
ENSMUSE00000643467		2:91013238-91019497		+		ENSMUST00000111452		Celf1		0.3755	0.709	0.612	0.8395	0.8565	0.846
ENSMUSE00001342001		1:165338188-165340023		−		ENSMUST00000193353		Dcaf6		0.082	0.1555	0.0585	0.0635	0.052	0.0535
ENSMUSE00000842895		11:106782469-106784018		−		ENSMUST00000133426		Ddx5		0.1375	0.1835	0.2265	0.3185	0.302	0.3625
ENSMUSE00000492954		3:95628541-95632102		+		ENSMUST00000037983		Ensa		0.458	0.6615	0.4515	0.4335	0.4195	NA
ENSMUSE00001357022		3:152213977-152215630		+		ENSMUST00000196062		Fubp1		0.0095	0.0535	0.058	0.0585	0.032	0.0515
ENSMUSE00000857219		1:161038225-161038539		+		ENSMUST00000160516		Gas5		0.2265	0.289	0.0485	0.068	0.1195	0.099
ENSMUSE00001326780		7:31134414-31135739		−		ENSMUST00000188032		Gramd1a		0.4465	0.7265	0.28	0.4175	0.319	0.307
ENSMUSE00000765273		11:50379468-50379964		+		ENSMUST00000134230		Hnrnph1		0.093	NA	0.2775	0.1575	0.181	0.165
ENSMUSE00000756514		X:95947770-95950446		−		ENSMUST00000126605		Las1l		0.0135	0.034	0.0545	0.041	0.043	0.0575
ENSMUSE00000764755		X:94537676-94538065		−		ENSMUST00000153386		Maged1		0.064	0.1	0.0455	0.0525	0.045	0.0545
ENSMUSE00000777868		5:21743379-21746090		+		ENSMUST00000125693		Pmpcb		0.0105	0.026	0.0865	0.033	0.0485	0.0665
ENSMUSE00000740654		X:8143848-8144679		−		ENSMUST00000141925		Rbm3		0.0675	0.0985	0.0195	0.0405	0.044	0.0695
ENSMUSE00001332031		1:55014483-55016490		−		ENSMUST00000187500		Sf3b1		0.1365	0.178	0.2265	0.264	0.284	0.282

Summary of the number of high confident Alternative Splicing (AS) events detected using rMATS pipeline (FDR <0.01) across developmental stages with replicates. Selected high confident exon skipping and intron retention events detected using rMATS pipeline (FDR <0.01) across developmental stages with replicates. Values across stages correspond to PSI values of the exons. Abbreviations used in the table stand for the following types of splicing events and definitions: SE- Skipped Exon, MXE- Mutually Exclusive Exon, RI- Retained Intron, A5SS- Alternative 5′ Splice Site, A3SS- Alternative 3′ Splice Site, PSI- Percent Spliced Index, FDR- False Discovery Rate.

summarizes the highly significant (1% FDR) SE and RI events discovered across developmental stages.

### Skipped exon events are the most abundant splicing events during lens development and are associated with differentiation, development and cytoskeletal regulatory pathways

Skipped exons are one of the most prevalent alternative splicing events in higher eukaryotes^39^. In these events, the splicing machinery can ‘skip over’ an exon by splicing it, thereby masking its contribution in the final RNA or protein product. We obtained 418 significant (FDR < 1%) exon skipping events corresponding to 266 exons observed in 399 transcripts from 213 genes across various developmental stages (Table S8).

**Figure 4:**
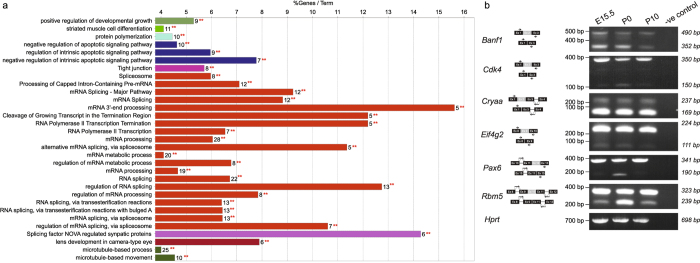
Functional analysis and validation of the high confident exon skipping events discovered across lens developmental states. (**a**) Functional enrichment analysis of genes associated with high confidence (FDR 1%) skipped exon events identified using rMATS^27^ pipeline in atleast one pairwise comparison of developmental stages. For each biological process per group (color coded), the % genes per GO term with number of query genes (** in red) in the analysis is shown in histogram. This shows the functional grouping of the GO-terms based on GO hierarchy using the Cytoscape^67^-ClueGO^31^ plugin. Significant clusters (p < 1e-2), color coded by group based on enriched GO-biological processes generated from ClueGO analysis with size of the nodes indicating level of significant association of genes per GO-term. (**b**) Experimental validation by RT-PCR analysis of a selected set of high confident skipped exonic events reveals that selected mRNA isoforms with skipped events are more abundant during embryonic and perinatal stages. The schematic of the expected products are shown next to the gene. For validation, primers (arrows) were designed on the exons (black box) flanking the alternatively spliced exon (grey box). For all the genes, band with higher molecular weight is the isoform including the alternatively spliced exon and band with lower molecular weight is the isoform with the skipped exon. *Hprt* represents a loading control. Negative control is included for all isoforms tested where the RT-PCR reaction was performed using the same primers as for the isoforms but without any cDNA. Full-length gels are included in Fig. S6.

We performed functional enrichment analysis of the genes associated with skipped exonic events identified at 1% FDR using ClueGO^31^ (Materials and Methods). Enrichment results from this analysis are summarized in Table S10. We found several significant (adjusted p-value < 2.05e-04) groups of functional processes to be enriched including ‘mRNA processing’, ‘microtubule-based process’, ‘splicing factor NOVA regulated synpatic proteins’, ‘regulation of intrinsic apoptotic signaling pathway’, ‘lens development in camera-type eye’, ‘protein polymerization’, ‘tight junction’, ‘positive regulation of developmental growth’ and ‘striated muscle cell differentiation’ (Table S10, Fig. 4a). These observations indicate the prevalence of skipped exonic events in several differentiation and developmental processes via post-transcriptional regulation. For instance, we found 6 genes significantly (adjusted p-value = 8.30e-05) associated with the term ‘lens development in camera-type eye’. The genes that belong to this functional theme include *Cdk4* (Cyclin-Dependent Kinase 4), *Cryba1* (Crystallin, Beta A1), *Lim2* (Lens Intrinsic Membrane Protein 2), *Meis1* (Meis Homeobox 1), *Pax6* (Paired Box 6), and *Smarca4* (SWI/SNF Related, Matrix Associated, Actin Dependent Regulator of Chromatin, Subfamily A, Member 4), which contributes to ~8% of genes annotated with lens developmental processes.

Paired Box 6 (*Pax6*) is a transcription factor encoded by 14 exonic gene *Pax6*. This gene has previously been documented as a key regulator for sensory developmental processes^40, 41^ and lens regeneration^42^. We found that a particular exon, ENSMUSE00001311933 (*Pax6*) was included in all developmental stages except P9 with high Percent Splicing Index (PSI) values ranging between 0.93 and 0.99. Similarly, we found that ENSMUSE00000736151 (Cyclin-Dependent Kinase 4, *Cdk4*) is differentially included in E18 (PSI value = 0.964) *versus* P0 (PSI value = 0.8025) (FDR < 1%) and ENSMUSE00000691476 (Crystallin, Beta A4, *Cryba4*) is included all developmental stages except P0 with high PSI values ranging between 0.97 and 0.99, suggesting its importance in lens development (FDR < 1%) (Table 3).

### Several skipped exonic events during lens development could be verified by RT-PCR and Sanger sequencing

We validated the expression of alternate isoforms of *Pax6* and *Cdk4* by RT-PCR and Sanger sequencing across developmental stages. Both *Pax6* and *Cdk4* follow the predicted trend (Table 3, Fig. 4b, Figures S7 and S8). For example, the ENSMUSE00001311933 exon of *Pax6* is expressed at stages E15.5 and P0, while its expression is undetected at P10. *Cdk4* exon ENSMUSE00000736151 is expressed at all three stages, E15.5, P0 and P10. Further, we validated skipped exonic events detected in four other genes (*Banf1*, *Cryaa*, *Eif4g2*, *Rbm5*) that have been detected at an FDR < 5% (Fig. 4b, Figure S7 and Table S8). Additional validation of these splicing events in P0 lens using Sanger sequencing independently confirmed our findings (Figure S8, Materials and Methods). Mutations in *Cryaa* have been previously shown to cause cataracts in humans and mice^43, 44^. *Eif4g2* and *Rbm5* encode for RNA binding proteins and *Banf1* encodes a DNA binding protein. While the function of these genes has not been characterized in the lens, they exhibit high expression in the lens tissue. Interestingly, another Rbm family protein, Rbm24, is expressed highly in vertebrate lens development^45^ and its deficiency in Zebrafish causes microphthalmia^46^. All five genes have alternatively spliced isoforms that are differentially expressed across lens developmental stages, as predicted by splicing analysis (Table S10). For example, the ENSMUSE00000145472 exon of *Banf1* is skipped at P10. Further, the ENSMUSE00000352893 exon of *Cryaa* is not highly expressed at any of the lens developmental stages tested, suggesting no potential function of the ENSMUST00000019192 transcript during late embryonic and early postnatal stages of mouse lens development. The isoform of *Eif4g2* containing exon ENSMUSE00000203223 is expressed in all developmental stages, while the alternate isoform without the exon is not as highly expressed. *Rbm5* has a distinct expression pattern during lens development. While the *Rbm5* isoform including the ENSMUSE00001225318 exon is expressed at all stages, the isoform with skipped ENSMUSE00001225318 exon is expressed highly only at P0. This suggests a potential function for the ENSMUSE00001225318 exon at early perinatal stages. Together, the RT-PCR validation analysis suggests that alternatively spliced isoforms of genes expressed in the lens are also differentially expressed at different developmental stages. This indicates that certain isoforms of genes function specifically during embryonic or postnatal development, indicating the significant contribution of post-transcriptional regulation to the functional diversity of the isoforms.

### Genes associated with retained intronic events are enriched for developmental check point, cellular response to stress and RNA-splicing regulators

**Figure 5:**
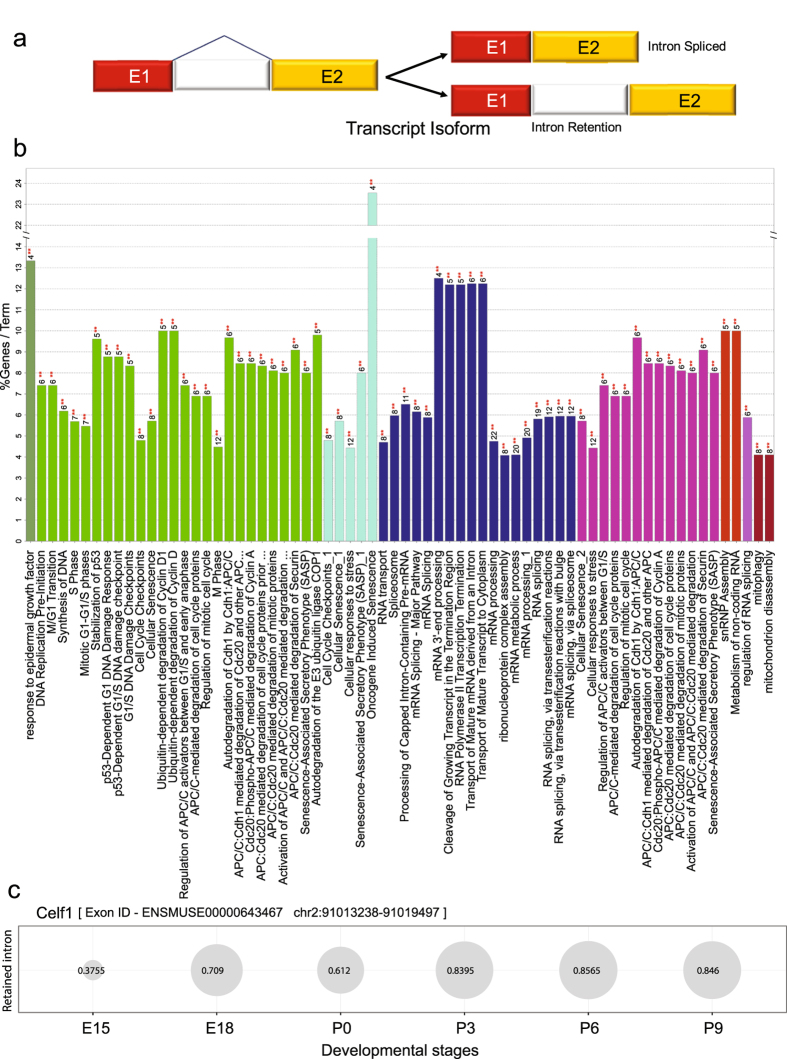
Functional analysis of the genes associated with high confident retained intron events across lens developmental stages. (**a**) Overview of intron retention mechanism (**b**) Functional enrichment analysis of genes associated with significant (FDR 1%) intron retention events identified using rMATS^27^ in atleast one pair of developmental stages compared. For each biological process per group (color coded), the % genes per GO term with number of query genes (** in red) in the analysis was shown in histogram. This shows the functional grouping of the GO-terms based on GO hierarchy was represented as Clustered GO-network using the Cytoscape^67^-ClueGO^31^ plugin. Significant clusters (p < 1e-2), color coded by group based on enriched GO-biological processes generated from ClueGO analysis with size of the nodes indicating level of significant association of genes per GO-term. (**c**) Bubble plot showing the alterations in the inclusion levels of a retained intron for Celf1 across various developmental stages. Each bubble shows the Percent Spliced Index (PSI) of the retained intron indicating an increase in the inclusion level from embryonic to post-natal stages.

Retained intron (RI) is an important but less characterized AS mechanism. It causes retention of intronic region that may or may not also include some exonic regions during splicing (Fig. 5a). It is commonly suggested that, most of the transcripts exhibiting RI, could open a new targeting motif for small interfering RNA (siRNA) at RI loci, thus are degraded by nonsense-mediated decay^47^. However, recent studies indicate that intron-retaining mRNAs are likely to have a more conserved role in development and numerous diseases^48^. Our splicing analysis indicated that retained intron events are the second most abundant alternative splicing events after skipped exon events (Table 3). We obtained 193 significant (FDR < 1%) intron retention events corresponding to 178 exons observed in 192 transcripts from 168 genes across various developmental stages (Table S9, Table 3).

Functional enrichment analysis of the genes which exhibited retained intronic events at 1% FDR threshold clearly revealed an enrichment for genes annotated with significant groups (p-value < 2.25e-04) such as ‘RNA splicing’, ‘M Phase’, ‘cellular responses to stress’, ‘autodegradation of *Cdh1* by *Cdh1:APC/C*’, ‘regulation of RNA splicing’, ‘snRNP assembly’, ‘response to epidermal growth factor’ and ‘mitophagy’ suggesting that the genes whose regulation is controlled by intron retention appear to be associated with developmental check points or stress related (Fig. 5b and Table S11). For instance, we found several genes (*Anapc2, Anapc5, Cdk4, Ehmt2, Ensa, H3f3b, Id1, Mcm7, Ncapg, Nup35, Pole, Ppp1cc, Psmc4, Psmd11, Psmd4, Rps27a, Tpr and Trp53*) associated with cell cycle [M-Phase], which were found to be exhibiting retained introns in various developmental stages (Table S11). Similarly, we found genes associated with ‘autodegradation of Cdh1 by Cdh1: APC/C’ to be significantly enriched (p-value = 1.87e-07) with 15 genes (*Anapc2, Anapc5, Atg4b, Becn1, Cdk4, Ehmt2, H3f3b, Id1, Map1lc3b, Psmc4, Psmd11, Psmd4, Rps27a, Trp53, Wipi2*) contributing to 6% of the genes associated with *Cdh1* mediated proteolysis/ degradation of mitotic proteins. *Cdh1* (epithelial cadherin) is an important protein which controls the mitotic arrest with G1-phase elongation in neurogenesis^49^.

*Celf1* (also known as CUG triplet repeat, RNA binding protein 1) is a well characterized RNA binding protein belonging to the CUG-BP family. CUG-BP family is known for protein members, which control the embryonically lethal abnormal vision via potential involvement in developmentally regulated alternative splicing^50^. *Celf1* is a highly conserved RNA binding protein, involved in alternative splicing, polyadenylation, mRNA stability, and translation processes^51^. Additionally, *Celf1* was documented to potentially regulate the genes involved in embryonic heart muscle development^51, 52^ with some support for the role of these family members in lens development^53^. In our analysis, we found that the last exon - ENSMUSE00000643467 (*Celf1*) exhibited retained intronic event with high PSI values in post-natal developmental stages compared to embryonic stages, with a very low abundance in E15 as shown in Fig. 5c. In addition to *Celf1*, several other RBPs were found to exhibit intron retention events supporting the notion that RBPs can be regulated post-transcriptionally by non-sense mediated mRNA decay of the unproductive splicing isoforms which might harbor stop codons^54^. These observations suggest a stage dependent regulation of RBP’s transcript levels by auto-regulation at post transcriptional level, to fine tune the downstream post-transcriptional regulatory networks in lens development.

### Eye Splicer: an interactive web-based genome browser for visualizing alternative splicing events across lens developmental stages

To facilitate easy access to the discovered splicing events across lens developmental stages, we have set up an interactive web-based genome browser, Eye Splicer (accessible via http://www.iupui.edu/~sysbio/eye-splicer/) powered by Biodalliance JavaScript library that enables visualizing skipped exon and retained intron events across developmental stages as tracks. After we collected inclusion levels from rMATS, we converted these into BED formatted text files, which were further converted into BigBed files to make them suitable for loading into Eye Splicer (see Materials and Methods). Figure S9 shows a screenshot of Eye Splicer showing a skipped exon event for ENSMUSE00001072738 exon of *Srsf2* (serine/arginine-rich splicing factor 2) gene. In this figure, which is manually edited to fit it in a small area, the change from E15 to P9 can be seen as the height of the bars corresponding to the PSI values scaled from 0 to 1. These clickable bars for each developmental stage provide a pop up a table summarizing the corresponding inclusion levels for the particular event type. *Srsf2* belongs to a family of pre-mRNA splicing factors, and constitute the spliceosome complex with documented role during embryonic development^55^. Our results indicate that the inclusion of this exon is increasing towards the later developmental stages. While in E15 PSI value of the skipped exon is 0.0535, it becomes 0.245 by increasing 4.5 times.

## Discussion

In this study, we investigated the transcriptomic alterations and splicing events during lens formation (i.e. across different developmental stages; E15, E15.5, E18, P0, P3, P6 and P9), and constructed a molecular portrait of known and novel transcript isoforms in the mouse lens. Although samples from the developmental time point E15.5 originated from a different study and read length distribution compared to the rest of the datasets, comparison of the annotated gene expression profiles in the mouse genome between E15.5 and E15 stages revealed a significant correlation (Pearson R = 0.86, p < 2.2e-16), suggestive of significant similarity in the expression profiles of developmentally close time points, irrespective of the source and sequencing platform. In contrast, expression profiles from E18 and post-natal stages exhibited lower correlation with respect to E15.5 dataset, further confirming the quality and robustness of expression profiles to delineate the developmental stages. Although, increasing number of studies using RNA-sequencing protocols are able to generate a wild type control as part of their research projects leading to stage-specific developmental transcriptomes^56–58^, several issues need to be considered before employing them in large-scale meta-analysis studies, which can significantly improve the quality and number of high confidence predictions. For instance, several of these publicly available datasets are generated with single replicates, provide separate transcriptomes of epithelial and fiber cells as opposed to whole lens, are generated with differing read lengths and arise from different labs. Hence, future efforts to integrate the datasets should account for sample heterogeneity by normalizing the samples before expression quantification or modifications should be adopted in splicing prediction software to account for variable sequencing fragment lengths across datasets.

Classification of ~25% of the total transcripts defined as novel transcripts, into partially and completely novel transcript types (PNTs and CNTs) based on their extent of overlap with current annotations, allowed us to uncover the properties of these transcript sub-types. We found that the extent of novelty of the transcripts decreased significantly in post-natal lens stages compared to embryonic stages, suggesting the presence of several uncharacterized novel transcript forms expressed during early lens development. PNTs were found to exhibit significantly higher conservation as well as expression levels compared to both completely novel and known transcripts, across the developmental stages studied here. Functional analysis of PNTs suggested the prominent role of several processes such as neural system development, structural morphogenesis, protein localization, cell division and differentiation, important for lens development. Notably, majority of the CNTs were widely expressed across developmental stages albeit exhibiting significantly lower expression, conservation and length compared to partially novel transcripts. Nevertheless, ORF prediction on a subset of ~600 CNTs which are conserved across all the studied species indicated protein coding ability for at least 30% of these novel transcripts. We confirm the expression of several of these CNTs across lens developmental stages. Functional analysis of the genes exhibiting the most abundant alternative splicing events, namely skipped exon and retained intron events, revealed the enrichment of mRNA processing, apoptotic signaling pathways, protein polymerization, cell development and differentiation for the former and the enrichment of cell cycle processes, stress and splicing regulation for the later type of events. We found several genes such as *Banf1, Cdk4, Cryaa, Eif4g2, Pax6* and *Rbm5* that are associated with lens development, to exhibit skipped exonic events. We have validated the expression of different isoforms as well as novel genes in developing mouse lens by qRT-PCR. Further, we have developed a splicing browser ‘Eye Splicer’ to access and view developmentally altered splicing events in mouse lens. Together, this in-depth analysis provides a high-resolution architecture of the mouse lens transcriptome and provides a one-stop portal for furthering the understanding of splicing alterations during lens development.

## Materials and Methods

To obtain a comprehensive understanding of the transcriptome and splicing alterations across various stages of lens development, we collected multiple publicly available RNA-seq datasets corresponding to the raw RNA sequence reads of mouse lens from different developmental stages (Table 1). These datasets were aligned to the mouse reference genome, quantified for expression levels of known and novel transcripts as well as to investigate splicing alterations as illustrated in the workflow (Fig. 1). In the following sections, each of the major steps employed in processing and analysis are described in further detail.

### Datasets employed and quality filtering of RNA-seq samples

We collected the raw RNA sequence reads of different developmental stages (E15, E15.5, E18, P0, P3, P6 and P9 each with its biological replicate) of mouse lens from Gene Expression Omnibus (GEO)^59^ and European Nucleotide Archive (ENA)^60^. Table 1 shows the relevant source of the RNA-seq dataset along with several metrics resulting from the alignment of the reads to the reference genome. Briefly, we downloaded the single end datasets in FASTQ format using the SRA Toolkit (fastq-dump command), and the paired end datasets were directly downloaded from ENA. We ensured the quality of the aligned sequence reads to a minimum quality score of 20 for each sample using FASTX-Toolkit (http://hannonlab.cshl.edu/fastx_toolkit/index.html).

### Sequence alignment of quality filtered RNA-Seq reads using HISAT

HISAT^25^ (Hierarchical Indexing for Spliced Alignment of Transcripts) is a highly efficient alignment tool for aligning short reads from RNA sequencing experiments onto reference genome. We used HISAT with default parameters and setting the number of processors to 32, for rapidly aligning the quality filtered RNA sequence reads collected from different sources (See Table 1) against mouse reference genome mm10 annotation files. SAM (Sequence Alignment/Map) files obtained as outputs from HISAT were post processed using SAMtools (version 0.1.19)^61, 62^ for converting SAM to BAM (Binary Alignment/Map) followed by sorting the output BAM files. The sorted binary alignment files (sorted-BAM) obtained after post-processing were employed for further data processing i.e. quantification of expression levels of transcripts and splicing analysis.

### Transcript identification and quantification from the aligned RNA-seq datasets

We used StringTie (version 1.2.1)^26^ for identification and quantification of transcripts from the aligned RNA-Seq reads. StringTie is novel network flow algorithm based on fast and highly efficient assembler, to quantitate the transcripts of each genomic locus considering all possible multiple splice events. In addition to annotated transcripts, it can also provide the information of possible novel transcripts in each sample. Transcript level expression data quantified using StringTie were stored in GTF (Gene Transfer Format) providing expression levels for both known as well as novel transcripts against mouse reference genome (mm10-Mus_musculus.GRCm38.84.gtf). All the GTFs previously obtained for each sample were grouped and provided as an input for stringtie “merge” mode along with mouse reference genome (mm10-Mus_musculus.GRCm38.84.gtf). The merged GTF thus obtained was then utilized as reference annotation file in re-running StringTie with the sorted-BAM for the corresponding samples. As a result, we obtained a matrix of expression levels for 90689 transcripts (68166 annotated and 22523 novel transcripts) in the mouse genome. Known transcripts are defined as the transcripts whose genomic co-ordinates and annotations completely overlapped with those reported in Ensembl database^63^ for the mouse genome. In contrast, novel transcripts were defined as the transcripts that were exclusively predicted by StringTie and hence could overlap partially with already annotated exonic regions in the mouse genome. A quantification matrix was generated for lens transcriptome with respect to different developmental stages extracting the TPM (transcripts per million) values from StringTie outputs. This matrix was utilized for downstream analysis.

### Defining and investigating the novel transcripts across developmental stages

We calculated the proportion of known and novel transcripts for each RNA-seq sample with an expression threshold of TPM > 1.0 and averaged the values for corresponding replicates from each developmental stage. The obtained proportions were represented as a bar graph for each developmental stage. Similarly, we calculated the proportion of known and novel transcripts with varying expression thresholds (TPM > 0.5, >2 and >5) and represented as bar graphs to study the reproducibility of our observed trends.

To investigate the discovered novel transcripts for their extent of novelty with respect to the known transcript architectures documented in the mouse reference genome mm10, we mapped the length of the discovered transcript to annotated reference transcript coordinates and calculated a novelty score for each novel transcript by using the below formula,

$${\rm{Novelty}}\,{\rm{Score}}=(1-\frac{{\rm{length}}\,{\rm{overlapping}}\,{\rm{region}}}{{\rm{full}}\,{\rm{length}}\,{\rm{of}}\,{\rm{novel}}\,{\rm{transcript}}})\times {\rm{100}}$$

We examined the distribution of novelty score of novel transcripts in each developmental stage and represented it as a density plot. We performed K–S (Kolmogorov–Smirnov) test to investigate for statistically significant differences in the novelty score distributions between any pair of developmental stages. Based on prior calculations and distribution of novelty scores, we categorized the novel transcripts into two groups; partially novel transcripts (PNTs, novelty score < 70%) and completely novel transcripts (CNTs, novelty score ≥ 70%). We analyzed the expression levels of transcripts across all stages for each transcript group - known, partially annotated novel and completely novel transcripts and performed Wilcoxon rank sum test to study the distribution of expression levels between transcript groups for each developmental stage separately. These results were represented as box plots in supplementary material.

### RT-PCR analysis of CNTs

To validate the expression levels of novel transcripts discovered from RNA-Seq analysis, total RNA was extracted using a RNeasy Mini kit (Qiagen Inc, Valencia, CA) from microdissected C57Bl/6 mouse lenses at three stages, namely, embryonic day (E) 15.5, and post-natal day (P)0 and P10. Each of the three biological replicates at E15.5 comprised of six lenses, and at P0 and P10 comprised of two lenses. RNA was treated with RNase free DNase (Qiagen Inc #79254, Valencia, CA). cDNA was synthesized from 200 ng of total RNA, representing three biological replicates at each developmental stage using Bio-Rad iScript^TM^ cDNA Synthesis Kit (Bio-Rad Laboratories, Hercules, CA), and was used as a template in PCR analysis. Primers were designed for the exonic regions of four CNTs (Table S12). The PCR products were run on 1% agarose gel. Presence of specific bands at the expected size were indicative of transcript expression in the lens.

### Phylogenetic conservation of mouse lens transcriptome

Although some reports indicate that mouse lens is likely to have a diverse transcriptome, the evolutionary significance of the transcriptome is poorly understood. Hence to address this, we investigated the evolutionary conservation of the identified transcripts. Multiple sequence alignment of genomic loci across several genomes provides a comprehensive snapshot of the evolutionary conservation, which can act as a proxy for functional preservation of a selected region^64^. For instance, protein coding genomic loci were documented to be highly conserved across the genome than non-functional genomic loci^65^. We applied this technique to conjecture and identify novel transcripts which could be functionality active across large phylogenetic distances. We downloaded the phastCons scores^66^ from the UCSC Genome Browser for the complete mouse genome. PhastCons score employed in this study provides an estimate of the individual nucleotide level conservation, calculated based on multiple sequence alignment of 46 vertebrate genomes with respect to mouse reference genome mm10. It ranges from 0–1 with higher the score higher is the conservation of the individual nucleotide across the genomes. For this study, we utilized the available nucleotide resolution conservation score data for mm10 and calculated the phastCons score for each exon of the novel transcripts by averaging the per-base scores and then computed a representative conservation score for each transcript as the mean phastCons score of the exons representing the novel transcript. Final scores were analyzed for known (annotated) transcripts, PNTs and CNTs to compare their relative extents of conservation.

Since Gene Ontology (GO) based functional enrichment analysis can provide important clues about the functions and molecular processes predominantly associated with novel transcripts, we analyzed the Partially Novel Transcripts (PNTs) that shared majority (>70%) of their genomic region with known/annotated transcript containing genes to understand the likely functions associated with them. This involved filtering the PNTs with phastCons score (>0.8) to first identify highly conserved transcripts and using the resulting set of genes associated with these PNTs for downstream functional analysis. Functional enrichment analysis was performed with p-value threshold < 10^−10^ for collected genes using Cytoscape^67^-ClueGO^31^ plugin and was represented as a clustered GO network. Significant clustering of genes, color coded by annotation group, based on enriched GO biological processes were highlighted in these representations.

Transcripts belonging to the completely novel class share less than 30% of their genomic region with known transcripts. We hypothesized that completely novel transcripts with high conservation and expressed in at least one developmental stage could be active with uncharacterized function. Hence, we filtered the transcripts based on phastCons score (>0.8) and analyzed their expression pattern. Expression profiles normalized by their maximum expression level across stages for these highly conserved completely novel transcripts were hierarchically clustered using Cluster 3.0^68^ and visualized as a heatmap using Java Treeview^69^. Representative hierarchically clustered panels of transcripts expressed in only one specific developmental stage and in all developmental stages were shown separately. Novelty Score (NS) and phastCons Score (PS) indices for transcripts were shown as an additional scale bar in each heatmap.

We also investigated the distribution of the number of exons and length of the transcripts for known, partially novel and completely novel transcripts. We performed K–S (Kolmogorov-Smirnov) test to evaluate whether length distributions of transcripts significantly differ. Likewise, exon counts were also compared for these three categories of transcripts.

In order to identify high confident completely novel transcripts for potential experimental validation, we applied three simultaneous filters namely phastCons score, expression and transcript length. Briefly, these robust filters comprised of novelty score set to 100% and (a) 300 ≤ transcript length ≤ 10000; phastCons score > 0.95; and average transcript expression (across all developmental stage) > 5.0 TPM in at least four developmental stages to generate novel transcript predictions broadly expressed across developmental stages and (b) 300 ≤ transcript length ≤ 10000; phastCons score > 0.95; exhibits expression in at most two developmental stages to generate novel transcript predictions specifically expressed in particular developmental stages. Resulting sets of broadly expressed, highly conserved and 100% novel transcripts were selected for experimental validation and discussed in the results section.

### Analysis of differential alternative splicing

RNA-Seq data provides an opportunity to detect differential alternative splicing events across conditions. Since we have two replicates of RNA-seq for each developmental stage of mouse lens tissue resulting from the same sequencing platform, we applied rMATS (replicate Multivariate Analysis of Transcript Splicing)^27^ to identify differential alternative splicing (AS) events. rMATS provides a computational framework to identify all possible splicing events which are altered between two samples, by inspecting the status of exons/introns as they are included or excluded resulting from alternative splicing. We used sorted BAM (Binary Alignment/Map) files, obtained from aligning the raw RNA-seq datasets against the mouse reference genome using HISAT as discussed above, as input to rMATS by pairing with their corresponding replicates from each developmental stage. This allowed us to compare each pair of developmental stages for alterations in various splicing events. Since rMATS requires all input datasets to have the same read length, we excluded the dataset from E15.5 which had a different read length compared to others. Also, we have provided the GFF (General Feature Format) file downloaded from Ensembl (version 82, September 2015)^70^ as input to rMATS and have used the default thresholds for remaining options. Briefly, rMATS enabled us to analyze the inclusion/exclusion of target exons/introns contributing to different types of alternative splicing events, namely skipped exon (SE), alternative 5′ splice site (A5SS), alternative 3′ splice site (A3SS), mutually exclusive exons (MXE) and retained intron (RI), across any pair of developmental stages with replicates. An AS event is quantified based on the difference in the level of inclusion of an exon which is defined as the splice index or Percentage Splicing Index ($\psi \,score$) between two samples or conditions and ranges between 0 and 1. PSI represents the inclusion/exclusion of an exon for a transcript isoform considering all alternate possible isoforms. Reads aligning to the alternative exon or to its junctions with adjacent constitutive exons provide support for the inclusion isoform, whereas reads aligning to the junction between the adjacent constitutive exons support the exclusion isoform; the relative read density of these two sets forms the standard estimate of *ψ*. Significant differences in the values of *ψ* for an exon, between a pair of conditions compared to a null distribution indicate its differential abundance. We ran rMATS for all pairs of six developmental stages (E15, E18, P0, P3, P6 and P9) and generated a summary table with the number of different alternative splicing events that were detected below 1% FDR threshold (Table 3). Since skipped exon and retained intron events were the most abundant, we collected these events from raw rMATS outputs specifically those which are supported by reads that span splicing junctions and reads on target below 1% FDR. Functional enrichment analysis of genes belonging to these splicing events was performed using ClueGO^31^.

### Experimental validation of the skipped exons

To confirm splicing events during lens development, we selected genes based on their potential relevance to lens biology and which were predicted with less than 5% FDR in our splicing analysis. For alternative splicing analysis, primers (listed in Table S12) were designed on exons flanking the alternatively spliced exon (skipped exon) on either side. Total RNA from E15.5, P0 and P10 C57Bl/6 mouse lens was collected as described above. RNA was treated with RNase free DNase (Qiagen Inc #79254, Valencia, CA). 200ng of lens total RNA was used as template for cDNA synthesis using *in vitro* reverse transcription kit as described earlier and cDNA was used as a template for PCR reactions. The different splice isoforms were identified based on size differences of PCR products separated by 1% agarose gel electrophoresis. We further analyzed the PCR products obtained using RNA from P0 lens by Sanger sequencing. The different splice isoform DNA bands from the P0 lens samples were excised from the gel and subjected to DNA purification using Wizard® SV Gel and PCR Clean-Up System (Promega #A9281, Madison, WI). DNA isolated from specific splice isoforms was sequenced by Sanger sequencing method.

### Development of a splicing browser for studying splicing alterations across developmental stages

The abundant AS events that were detected in this study namely skipped exons and retained introns, were made available for visualization via Eye Splicer (http://www.iupui.edu/~sysbio/eye-splicer/), an interactive web-based splicing browser for studying splicing alterations in mouse lens. Eye Splicer is built using the JavaScript library from Biodalliance (http://www.biodalliance.org). As Biodalliance requires BED (Browser Extensible Data) or BigBed formatted input files, we preprocessed these tables into BED formatted text files and generated the corresponding BigBed files, which are the compressed version of BED files and hence suitable for the web using the UCSC tools^71^. Eye Splicer has a simple interface with the lists of genes that have exons alternatively spliced below 1% FDR for skipped exons and retained introns, shown on the left menu and an interactive genome browser on the right which allows the visualization of the exons of interest upon selection from the gene lists or upon search using its text field that supports coordinate based search or gene name/Ensembl ID based search. Any viewable section of the splicing browser, can be exported using the Export button as SVG (scalable vector graphics). Eye Splicer is freely available on http://www.iupui.edu/~sysbio/eye-splicer/ and can be accessed without any login requirement.

## Electronic supplementary material

Supplementary Figures, legends and Table legends

Supplementary Tables S1-12
