# Supplementary material for: Transcriptome analysis of developing lens reveals abundance of novel transcripts and extensive splicing alterations
Source: Sci Rep. 2017 Sep 14;7:11572. doi: 10.1038/s41598-017-10615-4 (PMC5599659; doi:10.1038/s41598-017-10615-4)
Supplement: Supplementary file 1 — Supplementary Figures, legends and Table legends [file 41598_2017_10615_MOESM1_ESM.pdf]

Transcriptome analysis of developing lens reveals abundance of novel transcripts and extensive splicing alterations

Rajneesh Srivastava[ψ1], Gungor Budak[ψ1], Soma Dash[2], Salil A. Lachke[2,3], Sarath Chandra Janga[1,4,5*]

[1]Department of Biohealth Informatics, School of Informatics and Computing, Indiana University Purdue University, 719 Indiana Ave Ste 319, Walker Plaza Building, Indianapolis, Indiana 46202
[2]Department of Biological Sciences, University of Delaware, Newark, DE 19716
[3]Center for Bioinformatics and Computational Biology, University of Delaware, Newark, DE 19716
[4]Center for Computational Biology and Bioinformatics, Indiana University School of Medicine, 5021 Health Information and Translational Sciences (HITS), 410 West 10th Street, Indianapolis, Indiana, 46202
[5]Department of Medical and Molecular Genetics, Indiana University School of Medicine, Medical Research and Library Building, 975 West Walnut Street, Indianapolis, Indiana, 46202

[ψ] *Both authors contributed equally*

Running Head: Identification of novel transcripts and alternative splicing events in mouse lens

Keywords: mouse lens, expression, transcript, alternative splicing, developmental stages

** Correspondence can be addressed to :*

Sarath Chandra Janga
School of Informatics and Computing
Indiana University Purdue University
719 Indiana Ave Ste 319
Indianapolis, Indiana 46202
Phone: 317-278-4147
Email: scjanga@iupui.edu

Supplementary Material

Content:
Supplementary Figures S1-9.
Supplementary Tables S1-12.

Supplementary Figure Legends

Figure S1. Histogram showing the proportion of known and novel transcripts identified across various lens developmental stages in mouse. Only transcripts exhibiting an expression higher than (**a**) 0.5 TPM (Transcripts Per Million reads sequenced) (**b**) 2 TPM (**c**) 5 TPM are considered in the respective plots.

Figure S2. Gene ontology enrichment based functional grouping using annotations for genes corresponding to the high confidence partially novel transcripts (PS <0.76). Functional enrichment analysis was performed using cytoscape - clueGO plugin. (**a**) For each biological process per group (color coded), the % genes per GO term with number of query genes (** in red) in the analysis is shown in histogram. (**b**) Functional grouping of the GO-terms based on GO hierarchy was represented as clustered GO-network using the Cytoscape - ClueGO plugin. Significant clustering ($p < 1e-10$) of genes (color coded by group) based on enriched GO-biological processes generated by ClueGO analysis with size of the nodes indicating level of significant association of genes per GO-term is shown.

Figure S3. Boxplots showing the log10 transformed expression levels, averaged for biological replicates, for known (#23121), partially novel (#4531) and completely novel (#4027) transcripts, organized by the developmental stage. Expression levels were log10 transformed. Statistical testing was performed for each pair of transcript groups using wilcoxon test and documented in Supplementary Table S4.

Figure S4. Heat map showing the normalized expression profile of 647 completely novel transcripts. Completely novel transcripts with high conservation score (PhastCons Score > 0.8), and expressed in atleast one developmental stage are included. Expression profiles normalized by the maximum expression level of a transcript across developmental stages were hierarchically clustered using Cluster 3.0 and visualized as a heatmap using Java Treeview.

Figure S5. Analysis of the genomic structure of identified transcripts. (**a**) Histogram showing the distribution of the number of exons for known, partially novel and completely novel transcripts. (**b**) Kernel density distribution of transcript lengths (log10 transformed) for known, partially novel and completely novel. Statistical differences in the distributions of lengths were computed using the non-parametric Kolmogorov–Smirnov test on every pair of transcript types. Completely novel transcripts as a group were found to be significantly shorter than both partially novel and known transcripts. In contrast, partially novel transcripts were found to be longer than even the known transcripts.

Figure S6. This figure shows the full-length raw gel for RT-PCR analysis for validation of the expression of two CNTs with a predicted ORF (MSTRG.8249.1 and MSTRG.18685.1) and two

CNTs with no known ORF (MSTRG.17446.1 and MSTRG.21639.1) in E15.5, P0 and P10 lenses. *Hprt* represents a loading control.

Figure S7. This figure shows the full-length raw gel to confirm the experimental validation of exon skipping events by RT-PCR analysis of a selected set of high confident skipped exonic events, revealing that selected mRNA isoforms with skipped events are more abundant during embryonic and postnatal stages. For all the genes, band with higher molecular weight is the isoform including the alternatively spliced exon and band with lower molecular weight is the isoform with the skipped exon. *Hprt* represents a loading control.

Figure S8. Validation of the alternatively spliced products of *Banf1*, *Cdk4*, *Cryaa*, *Eif4g2*, *Rbm5* and *Pax6* by sanger sequencing. Sanger sequencing was performed on cDNA representing isoform-specific transcript regions amplified by RT-PCR from P0 lens and separated by gel electrophoresis. For each candidate gene, the longer isoform (larger sized product band) is considered to include an exon that is normally skipped in the shorter isoform (smaller sized product band). Chromatograms representing the exon-exon junctions of the longer (red) and shorter (green) isoforms are shown. A dotted line indicates exon-exon junction in each of the isoform sequenced. For example, sequencing chromatogram shows validation of exon 1 and exon 2 junction as well as exon 2 and exon 3 junction for the longer variant of Banf1 (left two chromatograms under the red line). Similarly, the shorter variant of Banf1 is validated by sequencing chromatogram that shows exon 1 and exon 3 junction (right chromatogram under the green line).

Figure S9. Screenshot from Eye Splicer (http://www.iupui.edu/~sysbio/eye-splicer/) showing the Percent Spliced Index (PSI) values for a skipped exonic event in Srsf2 gene across developmental stages. Inclusion levels of the exon were found to increase with developmental stage, suggestive of its usage in post-natal stages.

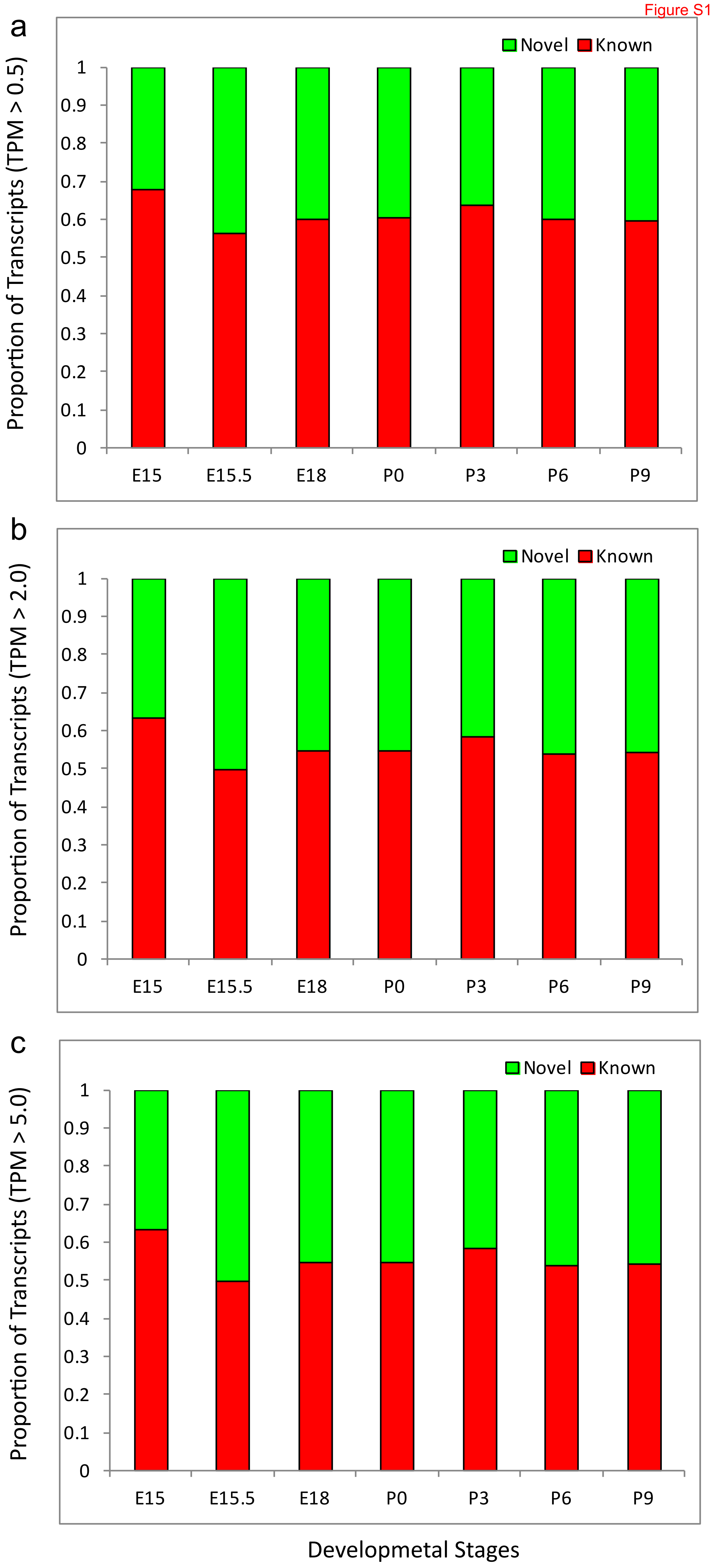
Figure S1
a
Novel
Known
Proportion of Transcripts (TPM > 0.5)
1
0.9
0.8
0.7
0.6
0.5
0.4
0.3
0.2
0.1
0
E15
E15.5
E18
P0
P3
P6
P9
b
Novel
Known
Proportion of Transcripts (TPM > 2.0)
c
Novel
Known
Proportion of Transcripts (TPM > 5.0)
Developmetal Stages

Figure S2

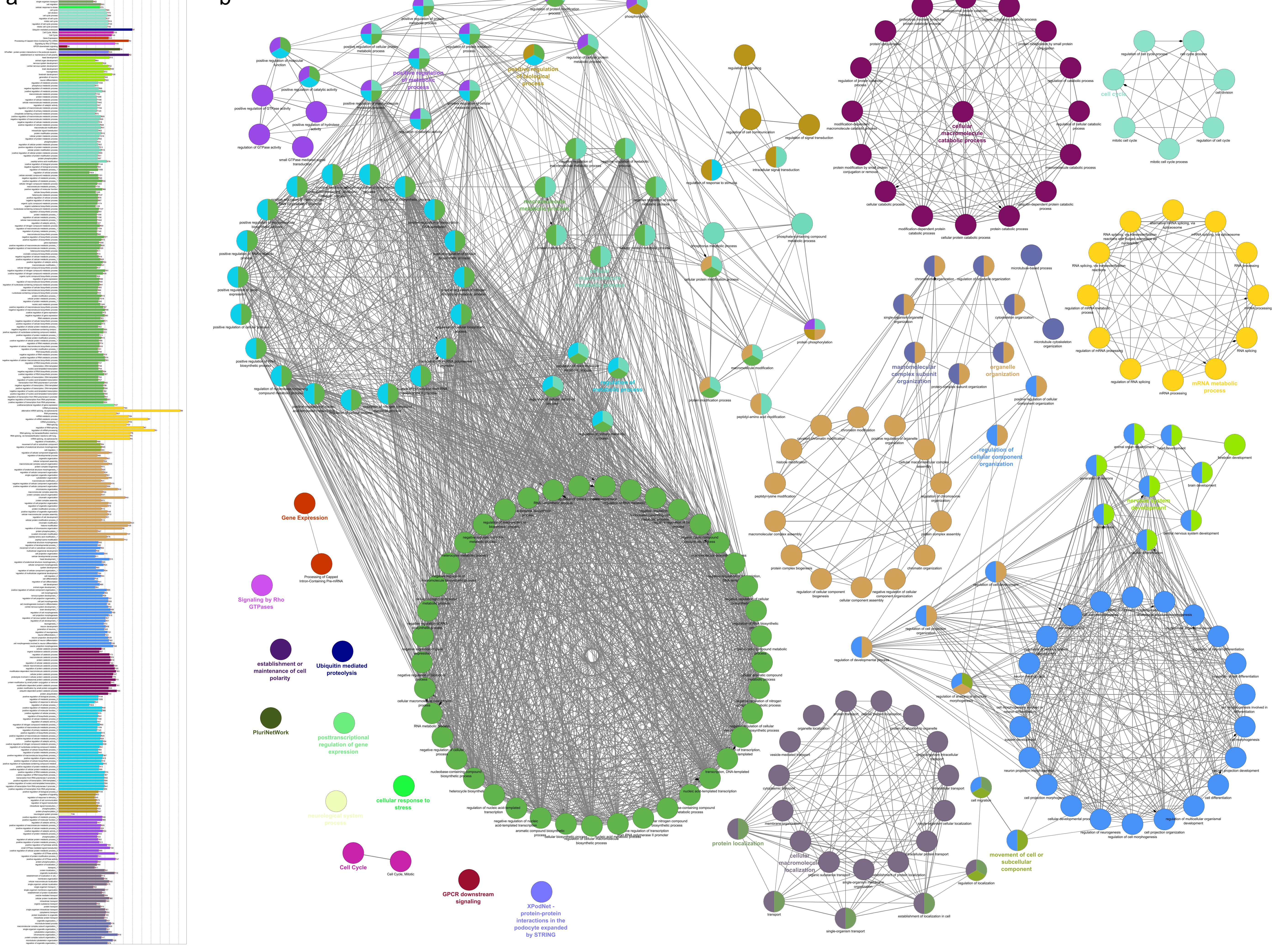

a
b
Gene Expression
Processing of Capped Intron-Containing Pre-mRNA
Signaling by Rho GTPases
establishment or maintenance of cell polarity
Ubiquitin mediated proteolysis
PluriNetWork
posttranscriptional regulation of gene expression
cellular response to stress
Cell Cycle
Cell Cycle, Mitotic
GPCR downstream signaling
XPodNet - protein-protein interactions in the podocyte expanded by STRING
positive regulation of metabolic process
positive regulation of biological process
macromolecule metabolic process
regulation of metabolic process
cellular macromolecule catabolic process
cell cycle
macromolecular complex subunit organization
organelle organization
mRNA metabolic process
regulation of cellular component organization
nervous system development
protein localization
cellular macromolecule localization
movement of cell or subcellular component

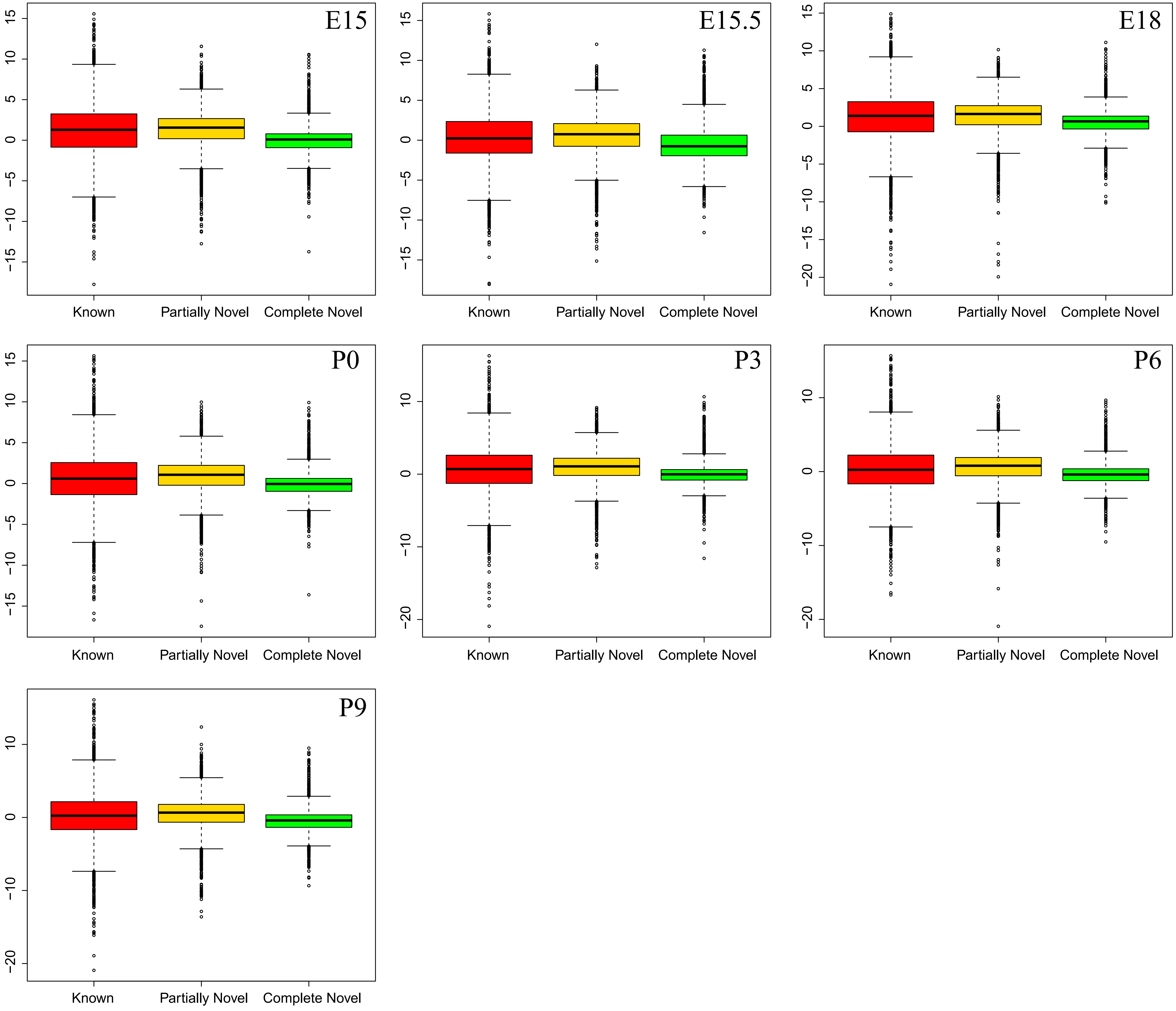
E15
E15.5
E18
P0
P3
P6
P9
Known
Partially Novel
Complete Novel

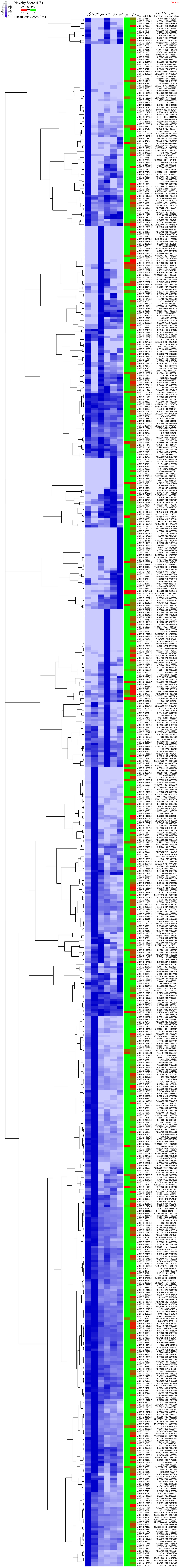
min
0.00
0.20
0.40
0.60
0.80
1.00
max
Avg.Expression (normalized by max)
Novelty Score (NS)
70 to 100
0.8 to 1.0
PhastCons Score (PS)
E15
E18
P0
P3
P6
P9
NS
PS
Transcript ID
mm10 Ref. genome Location (chr:start-stop)
MSTRG.7447.1 13:75954111-75954331
MSTRG.10714.1 16:88682165-88682703
MSTRG.12839.1 18:63543594-63543830
MSTRG.7392.1 13:69514010-69514236
MSTRG.29581.1 X:74493375-74493741
MSTRG.7110.1 13:50950369-50950599
MSTRG.8727.1 14:78966029-78966375
MSTRG.7309.1 13:63946209-63946411
MSTRG.28229.1 9:67747841-67748090
MSTRG.28042.1 9:57483177-57483565
MSTRG.10584.1 16:56021001-56021245
MSTRG.6777.2 13:15119006-15119437
MSTRG.16371.1 3:20474783-20475461
MSTRG.7901.1 14:14447656-14448019
MSTRG.1308.1 1:134395505-134395741
MSTRG.1823.1 1:176838584-176838833
MSTRG.7291.1 13:62967836-62968038
MSTRG.4236.1 11:64759412-64759711
MSTRG.26441.1 8:72283729-72284163
MSTRG.6067.1 12:69981028-69981767
MSTRG.13452.1 19:23166931-23168119
MSTRG.6241.1 12:82493274-82493813
MSTRG.23296.1 6:118474081-118474342
MSTRG.25152.1 7:107951379-107951776
MSTRG.12555.1 18:36649167-36649401
MSTRG.4045.1 11:56461459-56461672
MSTRG.440.21 1:51789248-51880097
MSTRG.6741.1 13:12259094-12259316
MSTRG.23360.1 6:122644265-122644770
MSTRG.5923.1 12:44621882-44622271
MSTRG.10567.1 16:53985023-53985331
MSTRG.10245.1 16:23050282-23050873
MSTRG.444.1 1:52585218-52585601
MSTRG.16203.1 2:177935577-177939007
MSTRG.27674.1 9:49079030-49079335
MSTRG.23694.1 7:3273749-3274023
MSTRG.26617.1 8:84854129-84854358
MSTRG.7902.1 14:14448146-14448740
MSTRG.18924.1 4:111807058-111807343
MSTRG.12432.1 18:25481526-25481854

Figure S5

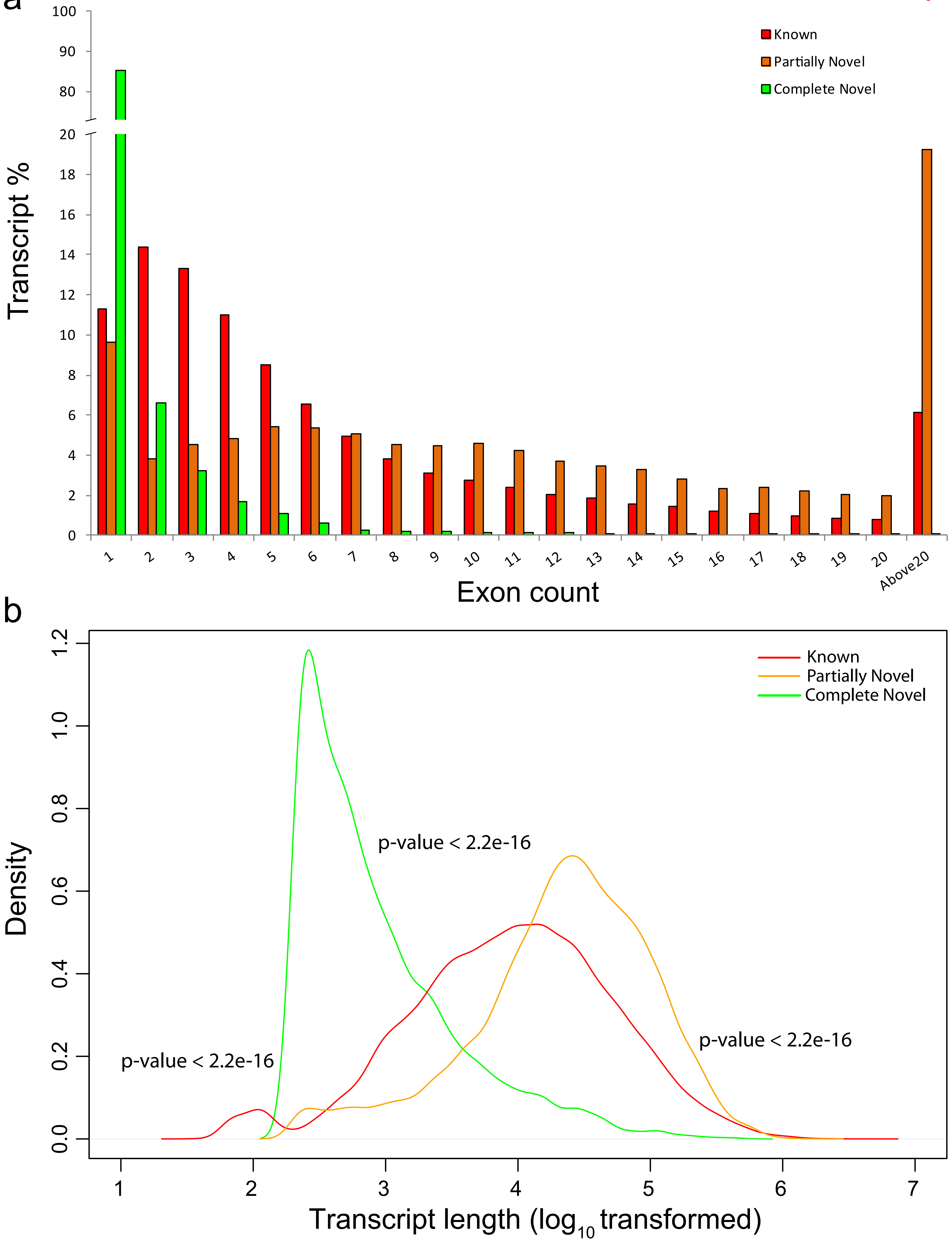
a
Transcript %
Known
Partially Novel
Complete Novel
Exon count
Above20
b
Density
Known
Partially Novel
Complete Novel
p-value < 2.2e-16
p-value < 2.2e-16
p-value < 2.2e-16
Transcript length (log10 transformed)

Figure S6

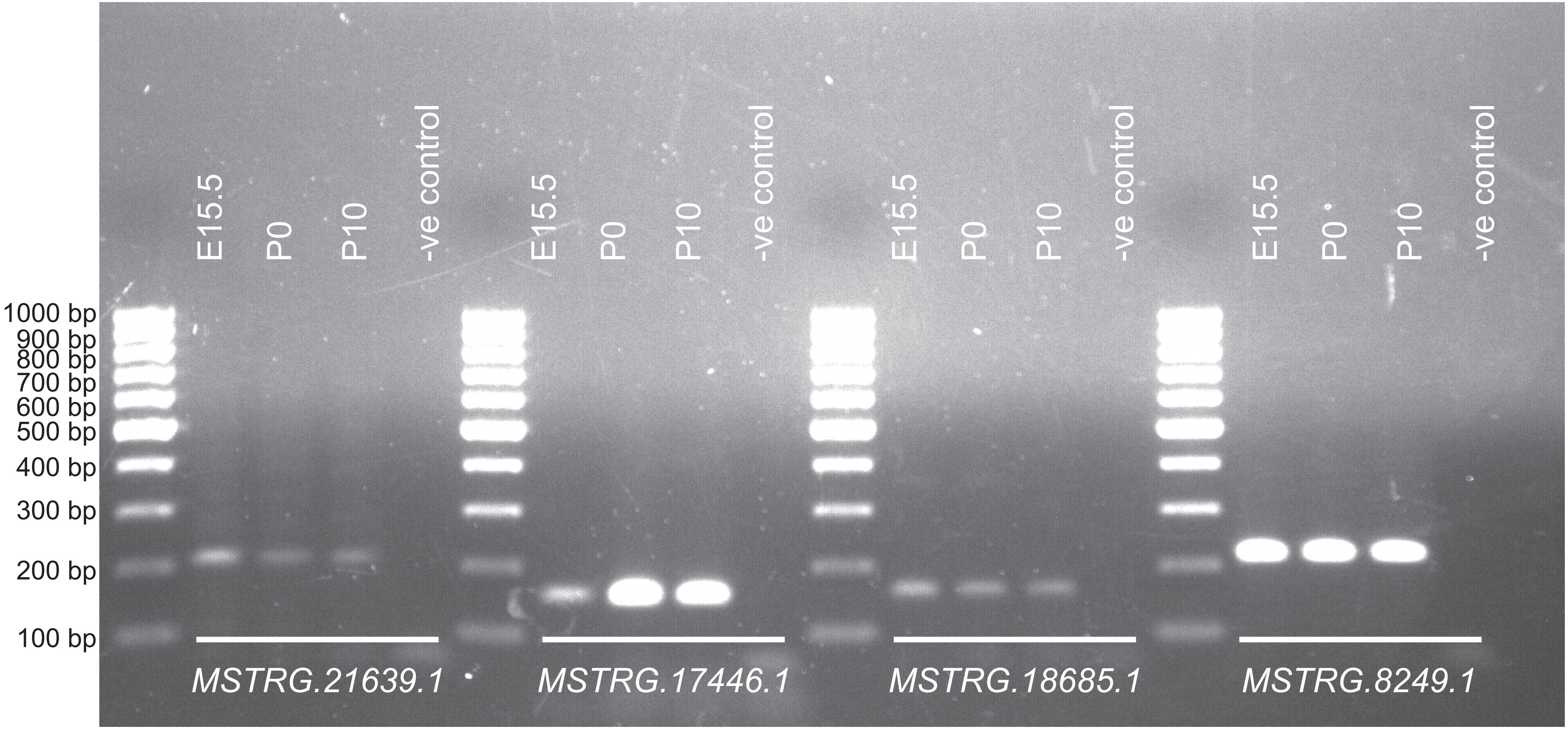
E15.5
P0
P10
-ve control
E15.5
P0
P10
-ve control
E15.5
P0
P10
-ve control
E15.5
P0
P10
-ve control
1000 bp
900 bp
800 bp
700 bp
600 bp
500 bp
400 bp
300 bp
200 bp
100 bp
MSTRG.21639.1
MSTRG.17446.1
MSTRG.18685.1
MSTRG.8249.1

Figure S7

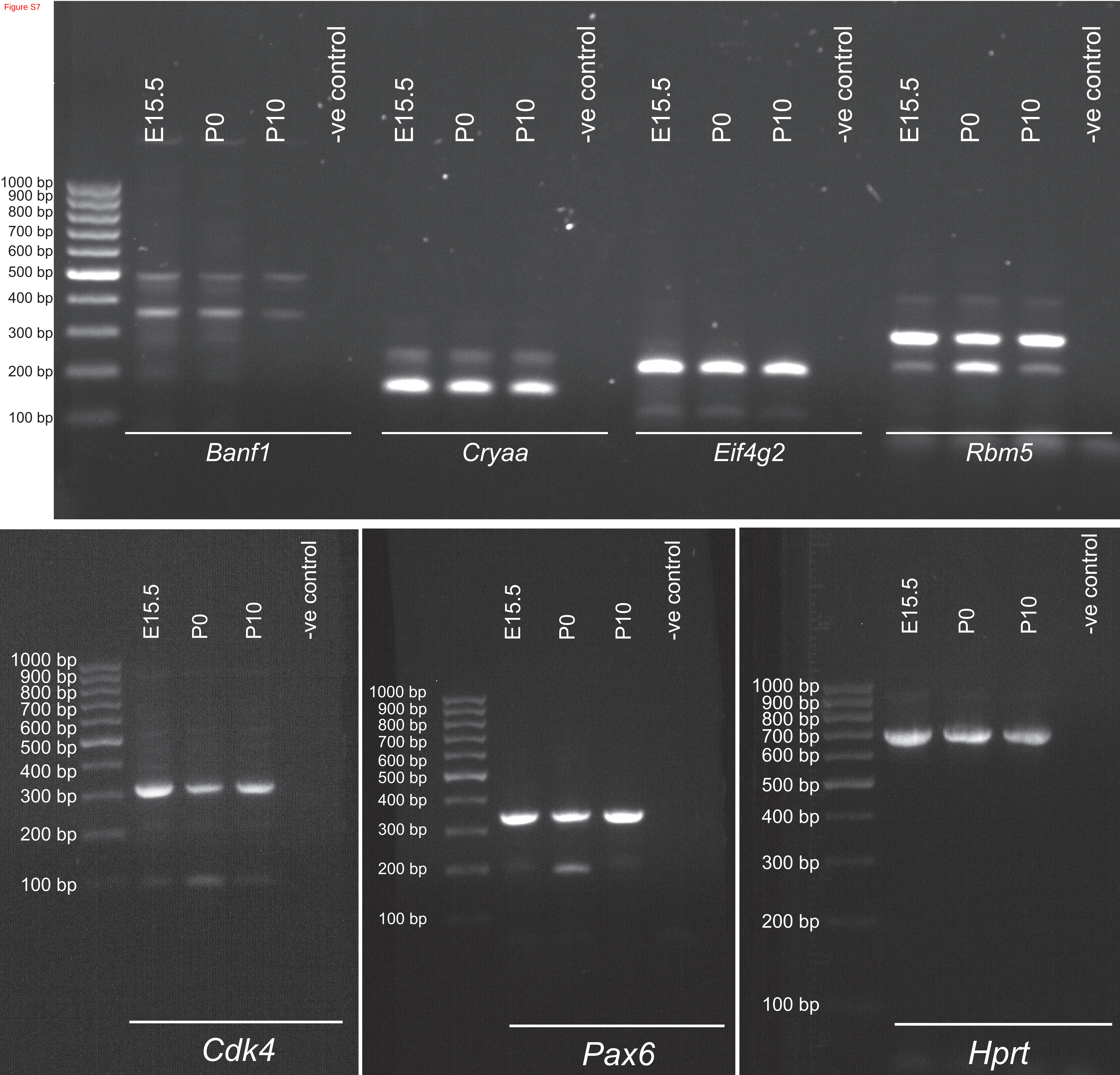
E15.5
P0
P10
-ve control
1000 bp
900 bp
800 bp
700 bp
600 bp
500 bp
400 bp
300 bp
200 bp
100 bp
Banf1
Cryaa
Eif4g2
Rbm5
Cdk4
Pax6
Hprt

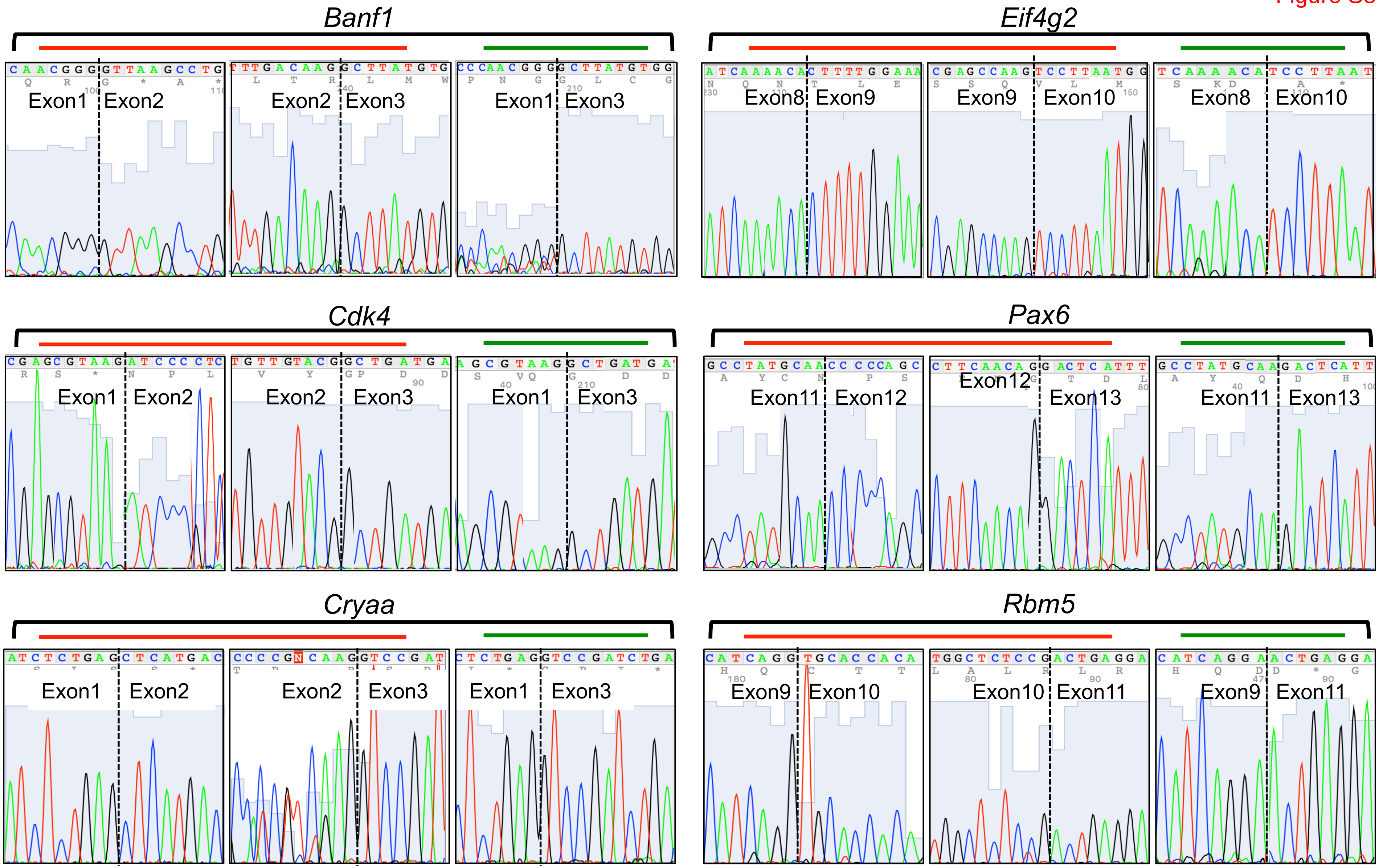
Banf1
Exon1 Exon2
Exon2 Exon3
Exon1 Exon3
Eif4g2
Exon8 Exon9
Exon9 Exon10
Exon8 Exon10
Cdk4
Exon1 Exon2
Exon2 Exon3
Exon1 Exon3
Pax6
Exon11 Exon12
Exon12 Exon13
Exon11 Exon13
Cryaa
Exon1 Exon2
Exon2 Exon3
Exon1 Exon3
Rbm5
Exon9 Exon10
Exon10 Exon11
Exon9 Exon11

Figure S8

Figure S9

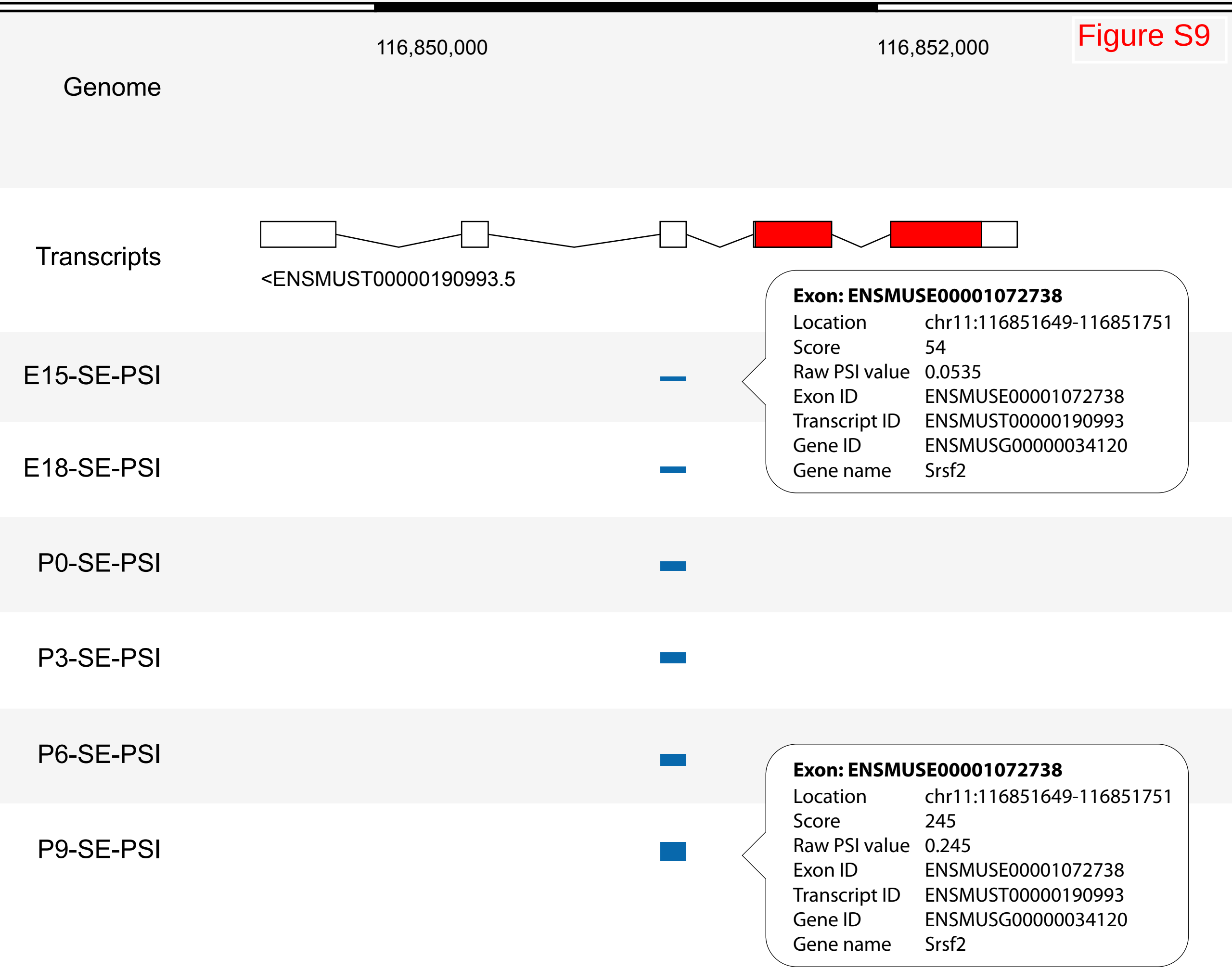

116,850,000
116,852,000
Genome
Transcripts
<ENSMUST00000190993.5
E15-SE-PSI
E18-SE-PSI
P0-SE-PSI
P3-SE-PSI
P6-SE-PSI
P9-SE-PSI
Exon: ENSMUSE00001072738
Location chr11:116851649-116851751
Score 54
Raw PSI value 0.0535
Exon ID ENSMUSE00001072738
Transcript ID ENSMUST00000190993
Gene ID ENSMUSG00000034120
Gene name Srsf2
Exon: ENSMUSE00001072738
Location chr11:116851649-116851751
Score 245
Raw PSI value 0.245
Exon ID ENSMUSE00001072738
Transcript ID ENSMUST00000190993
Gene ID ENSMUSG00000034120
Gene name Srsf2

Supplementary Table Legends

Table S1. Statistics of count and the proportion of known and novel transcripts identified across various lens developmental stages in mouse with varying expression (TPM - Transcripts Per Million reads sequenced) thresholds i.e. >0.5, >1.0, >2.0, >5.0.

Table S2. Expression profiles of detected novel transcripts along with their novelty score across developmental stages.

Table S3. Gene ontology enrichment based functional grouping using annotations for genes corresponding to the high confidence partially novel transcripts (phastCons Score > 0.76). Functional enrichment analysis was performed using cytoscape - clueGO plugin. Functional groups, associated genes and biological processes, with statistical significance, based on GO hierarchy are shown.

Table S4. The difference in expression levels, averaged for biological replicates, for known (#23121), partially novel (#4531) and completely novel (#4027) transcripts, was investigated using Wilcoxon test. Computed p-value for each pair of transcript classes for each developmental stage is documented.

Table S5. Expression profile of 647 completely novel transcripts with high conservation score (PhastCons Score > 0.8), and expressed in atleast one developmental stage was tabulated. This data is normalized and represented in Supplementary Fig. 4 as a heat map.

Table S6. Pfam domain predictions based on Open Reading Frames (ORFs) identified for CNTs which satisfied a) 300 bp ≤ transcript length ≤ 10000 bp; b) phastCons score > 0.95 and c) average expression > 5.0 TPM and expressed in at least four developmental stages. Out of 25 CNTs which satisfied these thresholds, 13 were predicted to have an ORF. Eight of these 13 ORFs were predicted to encode for documented Pfam domains.

Table S7. Pfam domain prediction based on ORFs identified in 654 CNTs which were 100% novel and exhibited a phastCons score > 0.8. Out of 202 CNTs that encoded for ORFs, we found 121 to exhibit at least one pfam domain using HMMSCAN.

Table S8. PSI (Percent Spliced Index) values of significant (FDR < 1%) set of exon skipping events across various developmental stages are shown.

Table S9. PSI (Percent Spliced Index) values of significant (FDR < 1%) set of intron retention events across various developmental stage are shown.

Table S10. Gene ontology enrichment based functional grouping using annotations for genes exhibiting the exon skipping events. Functional enrichment analysis was performed using cytoscape - clueGO plugin. Functional groups, associated genes and biological processes, with statistical significance, based on GO hierarchy are tabulated.

Table S11. Gene ontology enrichment based functional grouping using annotations for genes corresponding to exons exhibiting the intron retention events. Functional enrichment analysis was performed using cytoscape - clueGO plugin. Functional groups, associated genes and biological processes, with statistical significance, based on GO hierarchy are tabulated.

Table S12. Set of primers used for validating the CNTs and exon skipping events across lens developmental stages.
